# Is it possible to prevent excessive synaptic pruning in schizophrenia? Possibilities and limitations

**DOI:** 10.3389/fnsyn.2025.1656232

**Published:** 2025-10-15

**Authors:** Agnieszka Pawlak, Jakub Stefanowicz, Zofia Kotkowska, Agata Gabryelska, Marcin Sochal, Filip Napieraj, Magdalena Kotlicka-Antczak, Dominik Strzelecki

**Affiliations:** 1 Department of Affective and Psychotic Disorders, Medical University of Łódź, Łódź, Poland; 2 Department of Sleep Medicine and Metabolic Disorders, Medical University of Łódź, Łódź, Poland; 3 Medical University of Warsaw, Warsaw, Poland; 4 Department of Child and Adolescent Psychiatry, Medical University of Łódź, Łódź, Poland

**Keywords:** schizophrenia, synaptic pruning, microglia, C4A, BDNF, kynurenine pathway, neuroprotection, psychosis prevention

## Abstract

**Background:**

Synaptic pruning is a critical neurodevelopmental process that eliminates redundant or weak synaptic connections to optimize brain circuitry. In schizophrenia, converging evidence from imaging, genetic, and postmortem studies suggests that this process is pathologically accelerated, particularly in the prefrontal cortex during adolescence. The resulting reduction in synaptic density has been implicated in disrupted neural connectivity observed in psychosis, with the onset of cognitive impairment and negative symptoms.

**Objective:**

This review explores whether modulating aberrant synaptic pruning could serve as a preventive or early intervention strategy for schizophrenia. We analyze domains with emerging therapeutic relevance: tetracycline antibiotics, the complement system and C4 gene, kynurenine pathway modulation, epigenetic therapies, neuroprotective strategies (e.g., BDNF, NF-κB, progranulin), genetic and transcriptional regulators of pruning, and other new, mostly hypothetical, options. We also discuss the limitations of the impact on pruning.

**Methods:**

We conducted a structured review of the mechanisms involved in pruning, as well as clinical trials, preclinical studies, and mechanistic models that investigate molecular targets influencing synaptic pruning in schizophrenia.

**Results:**

Several molecular pathways have been implicated in abnormal synaptic pruning in schizophrenia, including complement C4A overexpression, kynurenine pathway imbalance (KYNA/QUIN), and dysregulation of microglial and transcriptional modulators such as MEF2C and TCF4. While retrospective studies suggest minocycline or doxycycline may reduce psychosis risk, randomized trials remain inconclusive. Emerging interventions, including LSD1 inhibitors, BDNF/progranulin enhancers, and lifestyle-based epigenetic modulation, show promise but require further validation in clinical settings. We also discuss the limitations of these methods, including safety considerations.

**Conclusion:**

Targeted modulation of synaptic pruning represents a promising but complex therapeutic strategy. The timing, specificity, and reversibility of interventions are crucial to avoid disrupting essential neurodevelopment. Future efforts should focus on identifying biomarkers for patient stratification and validating preventive strategies in high-risk populations.

## 1 Introduction

Recent advances in neuroscience have reinforced the hypothesis that schizophrenia is a neurodevelopmental disorder, with its roots tracing back to early brain maturation processes (Weinberger, 1987; Rapoport et al., 2012; Birnbaum and Weinberger, 2017). One of the most consistently implicated mechanisms is excessive synaptic pruning during adolescence, a period marked by widespread refinement of cortical circuits, particularly in the prefrontal cortex (Feinberg, 1982; Huttenlocher and Dabholkar, 1997). In individuals at risk for schizophrenia, this pruning appears to be hyperactive, leading to reduced synaptic density, disruptions in long-range connectivity, and impairments in information integration, all hallmark features of the illness observed in neuroimaging and postmortem studies, which have additionally underlined that decrease of dendritic spine density mainly refers to pyramidal neurons, particularly in layers III and V of the dorsolateral prefrontal cortex (DLPFC) (Glantz and Lewis, 2000; Kolluri et al., 2005; Glausier and Lewis, 2013; Sellgren et al., 2019). Significantly, this decrease is not associated with widespread neuronal loss, but rather with a reduction in neuropil volume, defined as the dense meshwork of dendrites, axons, and synaptic contacts that form the substrate of cortical connectivity (Glausier and Lewis, 2013; Konopaske et al., 2014).

This aberrant synaptic elimination is thought to be driven by genetic vulnerabilities, such as increased expression of complement component C4A (Sekar et al., 2016), as well as environmental factors that enhance neuroinflammatory signaling. Notably, pruning abnormalities may precede the onset of clinical symptoms by several years, offering a potential window for early intervention aimed at preserving neural connectivity and preventing negative symptoms and cognitive dysfunctions. Therefore, modulating the pruning process represents both a significant theoretical breakthrough and a therapeutic challenge in the future of personalized psychiatry. This raises a compelling clinical question: is it possible to selectively modulate synaptic pruning in a way that preserves healthy neural connectivity and reduces the likelihood of psychosis onset? The emerging literature suggests that multiple biological systems intersect with the pruning process, ranging from immune and inflammatory signaling, to metabolic and epigenetic regulation and offering potential targets for early therapeutic intervention. While interfering with synaptic pruning carries the risk of disrupting critical neurodevelopmental processes, advances in translational neuroscience and biomarker discovery have opened the door to more refined and reversible strategies. However, interfering with the physiological mechanisms of pruning could have significant consequences, so this approach must be taken with great caution. In this analysis, we review existing knowledge and discuss the possibilities of influencing the pruning process by modulating the various factors involved, with the aim of improving outcomes for patients with schizophrenia.

In order to obtain the most complete picture possible a comprehensive literature search for this review was conducted using major scientific databases including PubMed, Scopus, and Web of Science to identify articles related to synaptic pruning mechanisms and strategies aimed at preventing excessive synaptic pruning, particularly in the context of schizophrenia. The search included publications mostly from the past 20 years, with a focus on articles in English and available in full text. Search terms included combinations of: *synaptic pruning*, *excessive pruning*, *schizophrenia*, *microglia*, *complement system*, *preventive intervention*, *prognosis*, *neurodevelopment*, and *synaptic plasticity*. Initial results yielded over 900 articles; after screening titles and abstracts, 186 full-text articles were assessed as relevant for the review. Inclusion criteria involved articles that explicitly discussed the biological mechanisms of synaptic pruning, the role of pruning dysregulation in the pathogenesis of schizophrenia, and therapeutic or preventive strategies aimed at modulating pruning processes.

Interestingly, despite the significant importance of this topic for further research into effective methods of preventing and treating schizophrenia, only one article has attempted to synthesize the data linking the issues of interest to us here (Germann et al., 2021). However, we ensured that our work would expand on the themes raised in that article.

## 2 Mechanisms involved in pruning with modulation potential

**Figure 1:**
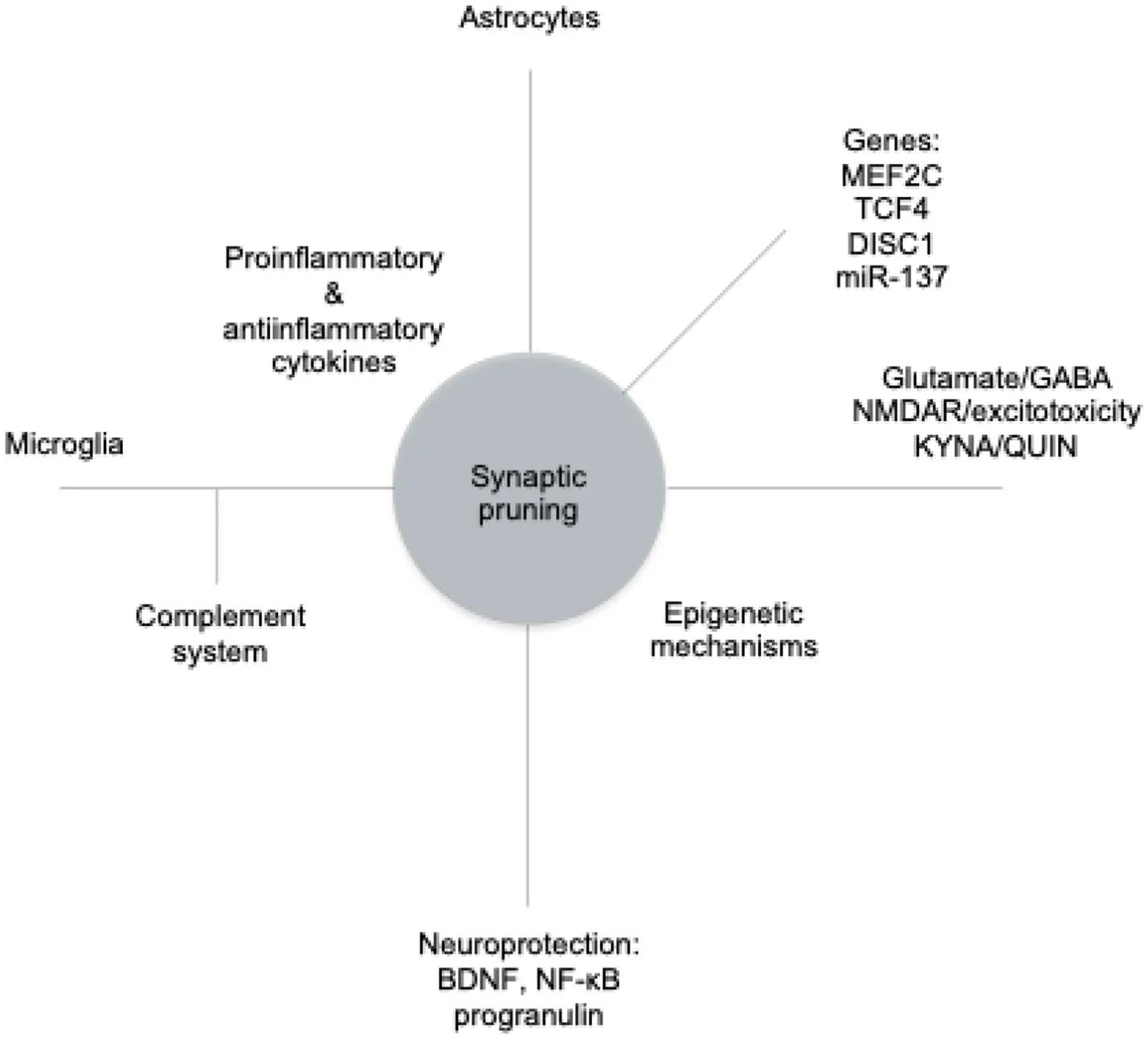
The main mechanisms modifying the pruning process. MEF2C, myocyte enhancer factor 2C; TCF, transcription factor 4; DISC1, disrupted-in-schizophrenia 1; miR-137, microRNA-137; GABA, gamma-aminobutyric acid; NMDAR, N-methyl-D-aspartate receptor; KYNA, kynurenic acid; QUIN, quinolinic acid; BDNF, brain-derived neurotrophic factor; NF-κB, nuclear factor kappa-light-chain-enhancer of activated B cells.

The Figure 1 illustrates the primary mechanisms involved in process modification, which we will discuss in detail below.

### 2.1 The complement system and the C4 gene

One of the most thoroughly studied biological pathways contributing to abnormal synaptic pruning in schizophrenia is the classical complement cascade. This pathway, a part of the innate immune system, as already mentioned plays a crucial role in marking synapses for elimination during brain development. Notably, the C4A isoform of the complement component 4 gene has been strongly implicated in this process. Groundbreaking research by Sekar et al. used postmortem brain tissue and genetic analyses to demonstrate that individuals with schizophrenia have increased copy numbers and higher expression levels of the C4A gene (Sekar et al., 2016). It leads to elevated production of complement proteins C1q and C3, which bind to synapses and mark them for phagocytosis by activated microglia, the brain’s resident immune cells, similar to how the immune system clears pathogens (Schafer et al., 2012; Hong et al., 2016a; Chen et al., 2025). The expression of complement proteins in neurons, particularly C1q, is regulated by astrocytes. Through this mechanism, astrocytes indirectly instruct microglia on which synaptic structures should undergo phagocytosis (Stevens et al., 2007). This crosstalk suggests that astrocytes serve as upstream regulators of microglial pruning activity. Mechanism of synaptic tagging and engulfment is essential for the developmental refinement of neural circuits, but it appears to be pathologically exaggerated in schizophrenia, particularly during adolescence when cortical pruning peaks (Mallya et al., 2019; Morini et al., 2021). Experimental models using human-derived microglia and induced pluripotent stem cell (iPSC)-derived neurons indicate that microglia from individuals with schizophrenia eliminate significantly more synapses than those from healthy controls. This hyperphagocytic phenotype is associated with increased C4A expression (Sellgren et al., 2019).

While the C4-complement–microglia axis is seen as a major contributor to synaptic deficits and cognitive dysfunction in schizophrenia, there are currently no clinical trials directly targeting C4A modulation (Ripke et al., 2014). However, indirect evidence suggests the pathological activation of this pathway in patients: multiple observational studies have reported elevated peripheral levels of C4 and C3 (Chen et al., 2021; Mohd Asyraf et al., 2022; Goker et al., 2023), also in ultra-high risk group (Laskaris et al., 2019).

Potential therapeutic strategies to counteract complement-mediated synapse elimination include inhibitors of complement proteins C1q or C3 (e.g., ANX005, a humanized anti-C1q antibody), monoclonal antibodies that block complement activation, and broad-spectrum microglial modulators like minocycline, which demonstrated minocycline has demonstrated anti-inflammatory and anti-phagocytic effects in both preclinical and early clinical studies (Miyaoka et al., 2008; Yang et al., 2022). Although many of these approaches remain largely preclinical or repurposed, they hold promise for selectively reducing hyperactive pruning without globally suppressing essential immune functions—an important consideration given the developmental significance of complement signaling.

### 2.2 Kynurenine pathway

The kynurenine pathway, a major route of tryptophan metabolism, plays a pivotal role in neurodevelopment, neurotransmission, and immune regulation (Pedraz-Petrozzi et al., 2020; Chen et al., 2024). Among its metabolites, kynurenic acid (KYNA) and quinolinic acid (QUIN) exert opposing effects on glutamatergic signaling and microglial activity, both of which are implicated in the pathophysiology of schizophrenia and particularly in synaptic pruning abnormalities (Schwarcz et al., 2012; Plitman et al., 2017; Inam et al., 2023). KYNA is synthesized primarily in astrocytes via kynurenine aminotransferases (especially KAT-II) and functions as an endogenous antagonist of NMDA receptors at the glycine site and α7 nicotinic acetylcholine receptors (α7-nAChRs) (Schwarcz et al., 2012). At physiological concentrations, KYNA plays a neuroprotective role, mitigating excitotoxicity and oxidative stress. However, in schizophrenia, elevated KYNA levels have been consistently observed in cerebrospinal fluid and postmortem cortical tissue, especially in the prefrontal cortex (Erhardt et al., 2007; Linderholm et al., 2012; Schwarcz et al., 2012; Plitman et al., 2017). This excessive KYNA may contribute to glutamatergic hypofunction, cognitive impairment, and disruption of microglial-mediated synaptic pruning during adolescence (Wonodi and Schwarcz, 2011). In contrast, QUIN, primarily synthesized by activated microglia, is a potent NMDA receptor agonist that promotes neuroinflammation, excitotoxicity, and oxidative stress. In the context of schizophrenia, elevated QUIN may drive excessive synaptic elimination, particularly when the balance between KYNA and QUIN is dysregulated (de Bie et al., 2016). The KYNA: QUIN ratio is therefore considered a key indicator of the neuroprotective versus neurotoxic potential of kynurenine pathway metabolism (Schwarcz et al., 2012). A comprehensive meta-analysis synthesizing over 100 studies and 10,000 subjects found no significant difference in absolute KYNA levels between individuals with schizophrenia and controls (Marx et al., 2021). However, the study revealed a significantly reduced KYNA/kynurenine ratio, indicating a metabolic shift away from the neuroprotective KYNA branch and potentially toward the neurotoxic QUIN pathway. This pattern may help explain excessive synaptic pruning observed in schizophrenia, particularly in early stages of illness. Mentioned findings are supported by experimental evidence from animal models. In rodents, pharmacological inhibition of KAT-II lowers brain KYNA levels, improves cognitive performance, and normalizes synaptic architecture (Bortz et al., 2017; Chang et al., 2018; Pocivavsek et al., 2019). Clinically, the KYN-5356 compound, a selective KAT-II inhibitor developed by Kynexis, completed a Phase 1 study demonstrating good tolerability and pharmacokinetics in healthy volunteers. A Phase 2 trial in individuals with schizophrenia is planned for 2025 (Kynexis Therapeutics, 2024). In animal models, inhibiting KAT-II by BFF816 or PF-04859989 decreased KYNA levels and improved working memory and protect synaptic integrity by preventing abnormal microglial phagocytosis (Linderholm et al., 2012; Kozak et al., 2014; Pocivavsek et al., 2019; Orhan et al., 2023).

### 2.3 Epigenetic mechanisms and therapies

Epigenetic mechanisms are undergoing increasingly intensive research, including schizophrenia (Swathy and Banerjee, 2017). In general, the most important of these include DNA methylation, which usually leads to the silencing of gene expression, and histone modifications (e.g., acetylation, methylation), which affect chromatin accessibility and regulate transcription (Kouzarides, 2007). In addition, non-coding RNAs, especially microRNAs, play an important role in regulating gene expression at the post-transcriptional level (Thomas and Zakharenko, 2021). In the excessive pruning observed in schizophrenia, DNA methylation (e.g., of genes encoding neurotrophic and inflammatory factors) and histone demethylation by the LSD1 (lysine-specific demethylase 1) affect microglial activation and the regulation of genes involved in synapse elimination (Park et al., 2022). LSD1 modulates chromatin accessibility and the transcription of neuronal genes that are involved in synaptic development and inflammation. Vafidemstat, a selective LSD1 inhibitor of lysine-specific demethylase 1 (LSD1/KDM1A), which is currently being investigated for its potential antipsychotic and pro-cognitive effects, may enhance neuroplasticity, reduce microglial activation, and normalize excessive synaptic pruning, particularly in the early stages of psychosis. The EVOLUTION phase IIb clinical trial is currently assessing its efficacy and safety in patients with schizophrenia (Oryzon Genomics, 2021). In addition to pharmaceuticals, lifestyle-based epigenetic interventions also show promise. Regular physical activity promotes the expression of brain-derived neurotrophic factor (BDNF) through histone acetylation, which aids in synaptic growth and decreases vulnerability to neurodegeneration linked to pruning. Furthermore, adequate sleep, anti-inflammatory diets (rich in omega-3 fatty acids and polyphenols), and supportive social relationships have been associated with beneficial epigenetic modifications in genes related to stress responses and neuroplasticity (Hölzel et al., 2011; McEwen and Morrison, 2013; Kaliman et al., 2014). These non-pharmacological strategies may buffer against maladaptive epigenetic remodeling and reduce the risk of pathological pruning during critical periods of neurodevelopment; they also appear to be the safest and most easily implemented. While many of these interventions are still in preclinical or exploratory stages, their combined potential to influence synaptic stability and immune function through epigenetic regulation supports their consideration as complementary strategies for preventing or slowing the progression of schizophrenia. Preclinical studies using rodent models have provided compelling evidence that epigenetic mechanisms tightly regulate synaptic pruning during postnatal brain development. Dysregulation of key enzymes such as histone deacetylases (HDACs), DNA methyltransferases (DNMTs), and LSD1 has been shown to alter microglial activation, synapse elimination, and behavioral outcomes relevant to schizophrenia. For instance, conditional knockout of HDAC1 or HDAC2 in mice disrupts the maturation of prefrontal circuits and impairs synaptic refinement (Akbarian and Huang, 2009), while pharmacological HDAC inhibition can reverse social and cognitive deficits in neurodevelopmental models (Guan et al., 2009). Similarly, inhibition of LSD1, a histone demethylase enriched in neural progenitors and synaptic compartments, leads to preserved synaptic density and reduced microglial-mediated pruning in postnatal mice, particularly under inflammatory conditions (Rusconi et al., 2016).

Notably, early-life stress and maternal immune activation models exhibit excessive synaptic elimination in the prefrontal cortex, paralleled by altered expression of epigenetic regulators and increased microglial engulfment activity (Marchi et al., 2021). We also know that omega-3 deficiency intensifies pruning in mice (Madore et al., 2020). Epigenetic interventions in these models—such as administration of HDAC inhibitors (e.g., valproate, sodium butyrate), DNMT inhibitors (e.g., RG108), or LSD1 inhibitors (e.g., ORY-1001)-have shown promise in restoring synaptic integrity and preventing long-term behavioral abnormalities (Sun et al., 2016; Christopher et al., 2017). These findings suggest that targeting epigenetic pathways may normalize aberrant pruning dynamics and offer a protective effect during critical periods of neurodevelopment.

### 2.4 Neuroprotection

Modulation of the brain-derived neurotrophic factor (BDNF) pathway is perceived as a promising neuroprotective strategy for preventing schizophrenia-related brain changes. BDNF plays a critical role in neuronal survival, synaptogenesis, and activity-dependent plasticity (Toader et al., 2025). In schizophrenia, decreased BDNF levels have been consistently associated with cognitive deficits and negative symptoms (Green et al., 2011; Fernandes et al., 2015; Yang et al., 2019). Consequently, BDNF is often used as a surrogate biomarker in clinical trials assessing the efficacy of novel interventions. Importantly, BDNF signaling is functionally intertwined with the transcription factor NF-κB (nuclear factor kappa-light-chain-enhancer of activated B cells), forming a regulatory feedback loop essential for maintaining synaptic integrity. Upon binding to its TrkB receptor (tropomyosin receptor kinase B), BDNF activates downstream signaling cascades, particularly PI3K/Akt and MAPK/ERK (phosphatidylinositol 3-kinase/protein kinase B and mitogen-activated protein kinase/extracellular signal-regulated kinase, respectively), which converge on NF-κB activation. This, in turn, promotes the transcription of genes critical for neuronal survival and plasticity, including Bcl-2 and c-Fos (B-cell CLL/lymphoma 2 and BCL2 apoptosis regulator, respectively) (Yoshii and Constantine-Paton, 2007; Minichiello, 2009). Reciprocally, NF-κB can also induce BDNF expression, creating a homeostatic circuit that sustains neuroplasticity and resilience under both physiological and stress-related conditions (Kaltschmidt and Kaltschmidt, 2009). This regulatory system is also influenced by additional molecular factors. TrkB signaling modulates pruning via local synaptic activity and neurotrophin gradients, guiding the elimination or maintenance of synapses during adolescence (Johnstone and Mobley, 2020). Conversely, stress-activated pathways such as the c-Jun N-terminal kinase (JNK) cascade and the transcription factor p53 can counteract neurotrophic signaling. JNK is activated by oxidative stress and cytokines, promoting microglial reactivity and synapse elimination (Castro-Torres et al., 2024), while p53 expression in microglia has been shown to facilitate synaptic loss in early neuroinflammation (Jebelli et al., 2014). Under neuroinflammatory conditions, which are typical in early psychosis, this axis becomes particularly vulnerable. Activated microglia utilize NF-κB to upregulate key elements of the classical complement cascade (e.g., C1q and C3), leading to synaptic tagging and excessive pruning (Hong et al., 2016b). When BDNF levels are concurrently reduced, either due to genetic predisposition or environmental insults, this immune-mediated elimination of synapses remains unchecked, contributing to cortico-limbic circuit disintegration and progression toward psychosis (Mondelli et al., 2011; Popovic et al., 2019). Enhancing BDNF availability may thus buffer against such maladaptive pruning. During adolescence, when synaptic remodeling is very intense, BDNF levels can be increased through regular physical activity, sufficient sleep, stress reduction, and anti-inflammatory diets rich in omega-3 fatty acids and polyphenols such as curcumin and resveratrol (Aid et al., 2007; Gómez-Pinilla, 2008; Szuhany et al., 2015). These interventions also appear to normalize NF-κB activity and reduce microglial overactivation (Yu et al., 2018; Zhang et al., 2019; Liu et al., 2021). In parallel, pharmacological agents such as N-acetylcysteine (NAC), 7,8-dihydroxyflavone, epigallocatechin gallate, and alpha-lipoic acid provide antioxidant and neurotrophic support to maintain synaptic architecture (Seifert et al., 2010). In summary, the BDNF–NF-κB axis, interacting with TrkB, JNK, and p53 signaling pathways, constitutes a multidimensional target for safeguarding synaptic stability during vulnerable developmental windows. Its modulation through both lifestyle and pharmacological strategies holds promise for reducing schizophrenia risk. At this stage, the significance of non-pharmacological interventions should be particularly emphasized. Future research should focus on stratifying interventions based on molecular profiles and timing of neurodevelopmental stages, particularly in high-risk adolescents (Angelucci et al., 2005).

Proinflammatory cytokines are increasingly recognized as key regulators of microglial-mediated synaptic pruning, particularly during adolescence. Under physiological conditions, cytokines such as interleukins IL-1β, IL-6, and TNF-α (tumor necrosis factor alpha) participate in maintaining neural homeostasis. However, when elevated chronically or during sensitive developmental windows, these molecules can intensify microglial activation, upregulate complement components, and induce excessive synaptic elimination (Boulanger, 2009; Perry and Holmes, 2014).

In the adolescent brain, cytokines influence pruning through several interconnected mechanisms. Firstly, they enhance microglial phagocytic capacity, promoting the engulfment of tagged synapses via the classical complement cascade (C1q/C3), often through activation of the NF-κB signaling pathway (Hong et al., 2016a). Secondly, proinflammatory cytokines impair neurogenesis and promote neuronal apoptosis, compounding the synaptic loss observed in schizophrenia (Yirmiya and Goshen, 2011). Thirdly, cytokines modulate astrocyte function and glutamate uptake, indirectly affecting synaptic stability (Tilleux and Hermans, 2007; Ye et al., 2013; Haroon et al., 2017). Astrocytes also release a range of signaling molecules, including TGF-β, which has been shown to drive neuronal C1q expression and consequently synaptic refinement (Bialas and Stevens, 2013). In addition, astrocytic cytokines can exert either pro- or anti-inflammatory effects, modulating microglial activity and thereby influencing the extent of synaptic elimination.

Animal models of maternal immune activation (MIA), such as prenatal exposure to lipopolysaccharide (LPS) or poly(I: C), have shown that elevated cytokine levels in the offspring are associated with excessive synaptic pruning and schizophrenia-like behavioral phenotypes (Garay et al., 2013; Knuesel et al., 2014). Administration of cytokine inhibitors during adolescence, such as monoclonal antibodies against IL-6R or TNF-α, can mitigate synaptic loss and improve cognitive functions in rodent models (Missault et al., 2014; Choi et al., 2016).

In humans, elevated serum levels of IL-6 and TNF-α in ARMS group predict transition to full-blown schizophrenia, suggesting a pathophysiological role of systemic inflammation (Mondelli et al., 2017). Adjunctive treatment with anti-inflammatory agents such as celecoxib or aspirin has shown modest but reproducible effects in improving negative symptoms when combined with antipsychotics (Laan et al., 2010; Müller et al., 2010). Pilot studies involving tocilizumab (anti-IL-6R) have begun to explore its potential to modulate immune-mediated neural changes in schizophrenia (Khandaker et al., 2018). Although most cytokine-targeted interventions remain in experimental stages, these findings underscore their promise as modulators of aberrant neurodevelopmental trajectories in schizophrenia.

Progranulin (GRN) serves as a protective factor against excessive synaptic pruning by microglia (Cenik et al., 2012). A deficiency in GRN leads to hyperactivation of microglia, increased expression of proteins C1q and C3, and impaired synaptic elimination during the pruning process (Petkau and Leavitt, 2014; Paushter et al., 2018). Studies on mice with GRN haploinsufficiency show an excessive removal of synapses in the cortex, along with behavioral abnormalities that resemble frontotemporal dementia (Lui et al., 2016). Additionally, reduced GRN expression impairs synaptic plasticity and long-term potentiation (LTP); therefore, GRN deficits may be related to the pathophysiology of schizophrenia and autism (Martens et al., 2012; Petkau and Leavitt, 2014; Lui et al., 2016). GRN concentrations, can be increased through several methods. One of the most effective approaches is to inhibit sortilin (SORT1), a protein responsible for the endocytosis and degradation of GRN. Blocking this pathway increases GRN levels in both the plasma and the brain (Hu et al., 2010). Another strategy involves the use of epigenetic drugs, such as histone deacetylase inhibitors (HDACi) and DNA methyltransferase inhibitors (DNMTi), which enhance GRN gene expression at the transcriptional level (Arrant et al., 2018). Preclinical studies have yielded promising results with certain natural compounds, including curcumin and trehalose, which stabilize GRN or directly or indirectly influence its production (Holler et al., 2016; Zhou et al., 2021). Research is also being conducted on gene therapies, such as AAV-GRN (adeno-associated viral progranulin), and the correction of GRN mutations using CRISPR-Cas9 (clustered regularly-interspaced short palindromic repeats-associated protein 9), particularly in the context of frontotemporal dementia (FTD) (Raitano et al., 2015; Arrant et al., 2018; Amado et al., 2019). Additionally, certain signaling pathways, such as mTOR and inflammatory cytokines (TNF-α, IL-1β), may indirectly regulate GRN levels, paving the way for new therapeutic opportunities in diseases linked to excessive synaptic pruning (Paushter et al., 2018).

### 2.5 Genes, transcription factors, and gene regulators

#### 2.5.1 Microglial factors

PU.1 factor (also known as SPI1) is a crucial regulator of microglial function, influencing both phagocytosis and the activation of brain immune cells (Cakir et al., 2022). The factors SALL1, MEF2C, and MAFB (spalt like transcription factor 1, myocyte-specific enhancer factor 2C, MAF bZIP transcription factor B, respectively) play significant roles in defining the phenotype and identity of microglia and in controlling pruning activity by regulating the microglial transcriptome (Yeh and Ikezu, 2019). Additionally, MEF2C is known to regulate the expression of genes that contribute to synaptic stability (Harrington et al., 2016).

#### 2.5.2 Neuronal and neurodevelopmental factors

Transcription factor 4 (TCF4) factor, also known as TCF7L2, is linked to the genetic risk of schizophrenia and plays a role in neuronal development as well as the regulation of gene expression in response to Wnt signaling and neural development. The NPAS3 (neuronal PAS domain protein 3) protein, part of the bHLH-PAS (the basic helix–loop–helix-PER-ARNT-SIM) superfamily, is involved in neurogenesis and the development and function of the cortical reelin system. Disruptions in its function have been observed in mouse models that mirror schizophrenia symptoms (Bernier et al., 2014). Additionally, proteins TBR1 (T-box brain transcription factor 1), TCF4, and TOP3B (DNA topoisomerase III beta) are crucial for the differentiation of cortical neurons, the establishment of appropriate neuronal connections, and the maintenance of synaptic stability, with TBR1 specifically playing a key role in layer VI (Rosato et al., 2021). The protein product of the DISC1 (Disrupted-in-Schizophrenia 1) gene, while primarily recognized for its structural functions, is also involved in neuronal migration and synaptic plasticity, which influences the structure of neuronal circuits (Dahoun et al., 2017; Fu et al., 2020). Research using animal models emphasizes the important role of DISC1 in the communication between GABAergic interneurons and pyramidal neurons (Sauer and Bartos, 2022), while both *in vitro* and *in vivo* studies have demonstrated that mutations in DISC1 lead to cytoarchitectural abnormalities in the cerebral cortex (Kamiya et al., 2005; Norkett et al., 2016) or behavioral changes resembling those seen in schizophrenia (Hikida et al., 2007; Niwa et al., 2013). RBFOX-1 protein (RNA binding protein, fox-1 homolog), which regulates alternative mRNA splicing in neurons, is found to have mutations in some individuals with schizophrenia. These mutations affect the composition of NMDA receptors and synaptic plasticity (Gehman et al., 2011; O’Leary et al., 2022). Additionally, the expression of RBFOX1 was found to be decreased in the PFC of individuals with this psychosis (O’Leary et al., 2022), particularly in parvalbumin-positive (PV+) GABAergic interneurons, which are key coordinators of the synchronous firing of pyramidal glutamatergic neurons (Chung et al., 2024). Lastly, dysregulation of NR4A2, also known as nuclear receptor related 1 protein (NURR1), may indirectly impact synapse survival due to its role in maintaining the dopaminergic system in schizophrenia (Ancín et al., 2013; Corley et al., 2016). The NURR1 mutant mouse is suggested as a potential model for studying behavioral and molecular mechanisms associated with schizophrenia (Rojas et al., 2007).

#### 2.5.3 Astrocytic factors

Astrocytes are capable of directly engulfing synaptic elements through receptor-mediated mechanisms. Two key proteins-receptors, MEGF10 (multiple EGF-like domains 10) and MerTK (Mer tyrosine kinase), enable astrocytes to identify and remove superfluous synapses. Mouse models deficient in MEGF10 and MerTK display excessive synapse numbers and disrupted circuit organization, underscoring the importance of astrocyte-dependent pruning in sculpting neural networks (Chung et al., 2013).

## 3 Other research directions

### 3.1 Tetracycline antibiotics

Tetracycline antibiotics, especially minocycline, exhibit neuroprotective effects unrelated to their antibacterial properties and may influence synaptic pruning, a process crucial for brain maturation, while disturbed in schizophrenia, autism, and Alzheimer’s disease, among others. Table 1 summarizes the proposed mechanisms of minocycline's effect on the pruning process.

**Table 1:** The main mechanisms of action of minocycline that can reduce excessive pruning processes.

Mechanism of action	Description	Potential effects	Key references
Inhibition of microglia	Reduction of synaptic activation and phagocytosis	Protection of neuronal connections	Yrjänheikki et al. (1999); Tikka et al. (2001); Henry et al. (2009)
Inhibition of MMP-9	Reduction of synaptic degradation	Stabilization of the extracellular matrix	Brundula et al. (2002); Yong et al. (2004)
Anti-inflammatory action	Decrease of TNF-α and IL-1β levels	Reduction of inflammation in the brain	Kim and Suh (2009); Plane et al. (2010); Garrido-Mesa et al. (2013)
Antiapoptotic effects	Protection of neurons from cell death	Potential improvement in negative symptoms and cognitive function	Pardo et al. (2005); Hong et al. (2016b); Sekar et al. (2016)

MMP-9, metalloproteinase 9; TNF-α, tumor necrosis factor alpha; IL-1β, interleukin 1 beta.

Evidence from a large-scale, population-based cohort study indicates that adolescents treated with doxycycline or minocycline for non-psychiatric infections may experience altered long-term psychiatric outcomes. In this retrospective cohort study, Sellgren et al. analyzed national health register data from over 1,000,000 individuals in Sweden and identified those exposed to tetracycline-class antibiotics during adolescence (Sellgren et al., 2019). The analysis revealed that exposure to minocycline or doxycycline for at least 90 consecutive days between the ages of 13 and 18 was associated with a significantly reduced risk of developing psychosis (adjusted hazard ratio: −0.6). It suggests a possible protective effect against early neurodevelopmental disturbances involved in the pathogenesis of schizophrenia. From a mechanistic perspective, minocycline crosses the blood–brain barrier and exhibits anti-inflammatory, anti-apoptotic, and microglia-modulating properties. These biological actions may interfere with abnormal synaptic pruning, particularly the microglia-mediated engulfment of synapses, which has been implicated in the early stages of schizophrenia. One open-label clinical study investigated the efficacy of minocycline as an adjunctive treatment in patients with schizophrenia receiving stable antipsychotic therapy. Twenty-two participants were administered minocycline at a dose of 150 mg/day for 4 weeks. The study reported statistically significant improvements in positive, negative, and general psychopathology symptoms measured by the PANSS scale, with benefits persisting 4 weeks after discontinuation. The treatment was well tolerated, with no serious adverse effects reported. The authors suggest that minocycline’s therapeutic potential may be linked to its ability to inhibit microglial activation, suppress inducible nitric oxide synthase (iNOS), and reduce caspase activity (Miyaoka et al., 2008). To further investigate these effects in a clinical context, the BeneMin trial (Deakin et al., 2018) was conducted. This 12-month, randomized, double-blind, placebo-controlled study assessed the efficacy of adjunctive minocycline in individuals with recent-onset schizophrenia (illness duration of less than 5 years). Two hundred seven participants received either minocycline (starting at 200 mg/day, then 300 mg/day) or a placebo in addition to standard antipsychotic treatment. The primary outcome, reduction in negative symptoms measured by the PANSS negative symptom subscale, did not differ significantly between the groups. Additionally, secondary biomarker outcomes, including medial prefrontal cortex gray matter volume, fMRI-based dorsolateral prefrontal cortex activation, and plasma IL-6 concentration, showed no statistically significant changes attributable to minocycline. Therefore, the trial failed to replicate the earlier promising findings from open-label and pilot studies, casting doubt on the clinical utility of minocycline in established schizophrenia. The results of the clinical trial NCT02569307, which investigates the use of minocycline and/or omega-3 fatty acids as adjunctive treatments alongside standard clinical care for individuals with At-Risk Mental State (ARMS), have not yet been published. This randomized controlled trial aims to evaluate whether these agents can delay or reduce the incidence of transition to first-episode psychosis (FEP), potentially by modulating neuroinflammatory pathways and abnormal synaptic pruning processes believed to underlie early pathophysiological changes in schizophrenia (Mushtaq et al., 2015).

In studies on mouse models of intracerebral hemorrhage, minocycline significantly suppressed C1q/C3–CR3 complement system proteins in the lesion area. It reduced microglial activation, neuronal apoptosis, and brain edema while improving neurological function. This effect was dependent on complement pathway inhibition, resulting in neuroprotection in this model (Li et al., 2021). In a series of experiments conducted on rats during the postnatal and adolescent periods, researchers investigated the effects of minocycline on neurogenesis in the subventricular zone (SVZ) and synaptic pruning. Minocycline inhibited or disrupted the proliferation of precursor cells, impaired neurogenesis, differentiation, and migration of nerve cells, and increased neuronal apoptosis in the postnatal stage of development (Shigemoto-Mogami et al., 2014; Inta et al., 2017). This inhibition of pruning resembles the actions of NMDA receptor antagonists, which have been associated with neuronal developmental disorders and may disturb the balance of plasticity in very young mice (Inta et al., 2016). Then, it is worth noting that although minocycline generally protects the nervous system in adults, it may disrupt regular pruning during early development. In cases of hyperactive pruning linked to C4A overexpression in schizophrenia, minocycline could be beneficial, provided that the timing of the intervention and the molecular profile of the patients are appropriately matched (Inta et al., 2017).

### 3.2 N-acetylcysteine (NAC)

NAC is one of the best-studied adjunctive therapies in schizophrenia, with particular emphasis on its role in regulating pathological synaptic pruning. Its mechanisms of action include antioxidant, anti-inflammatory, and glutamatergic pathways, which collectively protect the neuropil from excessive synaptic elimination. NAC serves as a precursor of glutathione (GSH), the brain’s main intracellular antioxidant. Schizophrenia is associated with reduced glutathione levels in the prefrontal cortex and hippocampus, contributing to oxidative stress, dendritic damage, and increased synaptic vulnerability (Do et al., 2009; Steullet et al., 2016). By replenishing glutathione, NAC mitigates oxidative damage and thereby prevents excessive pruning of synaptic connections. Oxidative stress and inflammatory stimuli activate microglia, leading to heightened synaptic phagocytosis. NAC reduces microglial activation and decreases the expression of pro-inflammatory cytokines, thereby suppressing pathological pruning processes (Bitanihirwe and Woo, 2011). NAC also modulates the cystine/glutamate antiporter system (xCT), indirectly affecting NMDA receptor activity. This redox-sensitive mechanism may reduce NMDA hypofunction and restore excitatory-inhibitory balance, which is crucial for preventing maladaptive synaptic elimination (Berk et al., 2008). Clinical studies have shown that NAC improves negative and cognitive symptoms in schizophrenia, most likely by enhancing synaptic plasticity and neuroprotection (Berk et al., 2008; Conus et al., 2018). A 6-month study assessing NAC or placebo effects on functional connectivity (FC) between cingulate cortex regions in 20 patients in the early phase of psychosis and 74 controls showed that, compared with placebo, NAC supplementation increased FC between regions linked to positive symptoms and processing speed in early psychosis (Mullier et al., 2019). Animal studies support these findings, showing that NAC protects dendrites and spines from oxidative stress-induced degeneration (Steullet et al., 2016).

### 3.3 Other compounds with potential effects on synaptic pruning

In addition to NAC, several other compounds show potential in modulating pathological synaptic pruning through antioxidant, anti-inflammatory, and neuroplasticity-supporting actions.

#### 3.3.1 Sulforaphane

This natural isothiocyanate present in cruciferous vegetables, is a potent activator of the Nrf2–ARE (nuclear factor erythroid 2-related factor 2–antioxidant response element) pathway, which regulates the expression of antioxidant and detoxifying enzymes. Activation of this pathway increases glutathione synthesis, improves redox balance, and reduces oxidative stress in the brain. In animal models, sulforaphane reduced microglial activation and protected against dendritic degeneration. Moreover, small clinical studies in patients with schizophrenia reported that sulforaphane supplementation improved cognitive functions, possibly by protecting the neuropil from excessive pruning (Dickerson et al., 2021; Hei et al., 2022).

#### 3.3.2 Glutathione precursors

L-cysteine and various exogenous forms of glutathione (e.g., GSH esters, liposomal glutathione) constitute another group of potential interventions. They act by increasing substrate availability for glutathione synthesis, thereby supporting antioxidant mechanisms and reducing synaptic vulnerability to oxidative stress-induced pruning. Clinical data on their efficacy remain limited, but experimental findings indicate that enhancing redox systems may help preserve neuronal network integrity and counteract dendritic spine loss (Do et al., 2009).

#### 3.3.3 Omega-3 fatty acids

These fatty acids, especially eicosapentaenoic acid (EPA) and docosahexaenoic acid (DHA), exhibit potent anti-inflammatory and neuroprotective properties. Their actions include reducing pro-inflammatory cytokine levels (e.g., TNF-α, IL-6) and supporting the expression of neurotrophic factors such as BDNF, which is critical for synaptic plasticity. Omega-3 supplementation has been studied in individuals at ultra-high risk for psychosis and was shown to reduce the risk of transition to first-episode psychosis (Amminger et al., 2010). This effect relies on the protection of synapses from degradation caused by neuroinflammatory and oxidative processes linked to excessive pruning.

### 3.4 Endocannabinoid system modulators

Activation of CB₂ receptors, mainly present in microglia, has a neuroprotective effect, reducing synaptic phagocytosis and limiting inflammation in the CNS (Mecha et al., 2016; de Almeida and Martins-de-Souza, 2018; Ferranti and Foster, 2022). Cannabidiol (CBD) suppresses pro-inflammatory signaling pathways (e.g., NF-κB, STAT3) reducing microglia-mediated neuroinflammation and preserving neuronal integrity (Yousaf et al., 2022). CBD is being studied in the context of early schizophrenia as part of the CANGLIA study, which assesses the effect of CBD on microglia activation using proton MRI spectroscopy (Bossong, 2017; Rodrigues da Silva et al., 2025).

### 3.5 Gut-brain axis modulators

Short-chain fatty acids (SCFAs), such as butyrate, may influence microglial activation and neuroinflammatory gene expression, suggesting their potential involvement in pruning regulation (Valles-Colomer et al., 2019; Ju et al., 2023). Research indicates that in animal models, interventions to correct intestinal dysbiosis may alleviate pathological synaptic pruning by modulating microglial activity and the C3/CR3 complement pathway. In mice, chronic stress induced dysbiosis and excessive C3 activation, leading to synaptic loss, whereas restoration of normal microbiota inhibited this process (Hao et al., 2024). Supplementation with prebiotics, probiotics, or synbiotics reduced inflammation, improved microglial function, and preserved dendritic spine density (Chunchai et al., 2018). Fecal microbiota transplantation (FMT) from young individuals increased synaptic plasticity and reduced the aging phenotype of microglia (D’Amato et al., 2020).

### 3.6 Neurosteroids

Substances from this group, especially allopregnanolone (ALLO), have anti-inflammatory effects and counteract excessive microglial activation (Jolivel et al., 2021). ALLO inhibits activation of toll-like receptors (TLR2/TLR4) on microglia, thereby preserving a ramified, surveying morphology and reducing phagocytic activity that contributes to pathological synaptic pruning. In mouse and rat models, ALLO (3α,5α-THP) directly inhibits MyD88-dependent TLR4 signaling and diminishes expression of TLR4 and TLR7, leading to reduced production of pro-inflammatory cytokines and normalization of microglial morphology (Balan et al., 2021). *In vitro* studies using BV-2 (immortalized mouse-derived microglial cell line) and primary murine microglia demonstrate that ALLO induces extension of cellular processes and decreases phagocytosis, indicating its ability to oppose neuroinflammatory pruning (Jolivel et al., 2021).

Golexanolone, a novel GABA-A receptor modulating steroid antagonist, was found to inhibit microglial and astrocyte activation, normalize glial function, and maintain ramose microglial morphology, counteracting structural changes typical of neuroinflammation (Bäckström et al., 2024).

Etifoxine, a clinically used neurosteroid that acts as a Translocator Protein (TSPO) ligand, significantly reduces microglial activation in brain injury models, such as traumatic brain injury (TBI) in rats. This reduction in activation leads to decreased production of pro-inflammatory cytokines and offers protection to nerve cells from degeneration (Simon-O’Brien et al., 2016). The mechanism behind this effect is linked to TSPO activation, which promotes the synthesis of neurosteroids, including pregnenolone, progesterone, and allopregnanolone, which are powerful modulators of GABA-A and have properties that inhibit inflammation and neurodegeneration (Rupprecht et al., 2010, 2023). Additionally, etifoxine has been observed to improve functional and cognitive outcomes in TBI models. This improvement is associated with enhanced mitochondrial homeostasis and reduced microglial activation, mechanisms that are both crucial for protecting synapses during pathological pruning (Palzur et al., 2021). In animal models of CNS injury progesterone reduces levels of inflammatory metabolites of C3 and proinflammatory cytokines (IL-1β, TNF-α) and may have a protective effect on synaptic structure (Pettus et al., 2005; Zhou et al., 2024).

Although none of the above approaches has yet undergone full clinical validation in the context of schizophrenia, their mechanisms of action suggest the potential for selective modulation of pruning and reduction of neurodegeneration. Further translational research and the determination of a safe therapeutic window are needed.

## 4 Emerging strategies in preventing maladaptive synaptic pruning

Beyond pharmacological interventions, several novel and still largely experimental strategies are under investigation:

### 4.1 Microglia-targeted immunotherapy

Microglia play a central role in pruning through CR3 (complement receptor 3), which recognizes complement-opsonized synapses and triggers their phagocytosis (Schafer et al., 2012). Experimental blockade of the CR3 signaling pathway reduced synapse elimination in animal models, suggesting that immunotherapies targeting microglial activity may represent a strategy to preserve synaptic connectivity in schizophrenia (Hong et al., 2016a).

### 4.2 Cellular therapies

Patient-derived induced pluripotent stem cells (iPSCs) allow *in vitro* modeling of pathological pruning and neuron–microglia interactions. It enables the study of individual genetic variants (e.g., C4) and testing of candidate drugs under biologically relevant conditions (Sellgren et al., 2019).

### 4.3 Optogenetics and chemogenetics

Advanced tools such as optogenetics and chemogenetics (designer receptors exclusively activated by designer drugs, DREADDs) permit selective and reversible control of microglial and neuronal activity in animal models. These methods allow precise dissection of causal links between microglial activation and synaptic elimination, paving the way for potential future interventions targeting specific cell populations (Yeh et al., 2017; Bolton et al., 2022; Roux et al., 2023).

### 4.4 Nanotechnology in drug delivery

Microglia-targeted nanoparticles and nanocarriers for anti-inflammatory or antioxidant drugs represent an innovative strategy to precisely deliver therapeutic agents to cortical regions most vulnerable to excessive pruning. This approach minimizes systemic side effects while enhancing local neuroprotection, making it a promising translational direction (Zhao et al., 2020; Battaglini et al., 2024).

### 4.5 Neuropeptides

Increasing attention is focusing on neuropeptides regulating sleep, mood, and social interactions, such as orexin and oxytocin. Evidence suggests that they modulate microglial activity and support synaptic plasticity, indirectly influencing pruning. Neuropeptide-based interventions may help restore neuroimmune balance and improve neuronal network function in schizophrenia (Makinodan et al., 2012; Yuan et al., 2016; Selles et al., 2023).

## 5 Discussion

### 5.1 Early identification of high-risk individuals

A family history of schizophrenia, affective disorders, or psychotic spectrum disorders is one of the most important risk factors for excessive synaptic pruning. Early identification of such individuals allows them to be included in monitoring and prevention programs even before the first psychotic symptoms appear (Feinberg, 1982). Detection of prodromal symptoms and windows of plasticity through the recognition of subtle changes in cognitive function, sleep, mood, or social interactions in individuals with a family history provides an opportunity for intervention at a time when brain plasticity remains high and the pruning process is potentially modifiable (Germann et al., 2021). In addition, individuals with a family history may undergo imaging tests, such as magnetic resonance imaging, to assess cortical volume or gray matter density, as well as analysis of neuroinflammatory biomarkers in body fluids. Early detection of such abnormalities provides an opportunity to take preventive measures before structural changes in the brain become permanent (Cannon et al., 2015). In individuals with a family history of schizophrenia, the selection of strategies to minimize factors that exacerbate pruning should be of particular importance. Data from previous studies indicate the possibility of implementation of stress reduction methods, improving sleep quality, modulating the gut microbiota (probiotics, diet), and avoiding psychoactive substances such as THC or amphetamines (Germann et al., 2021). As mentioned, early use of anti-inflammatory and microglial-modulating drugs, such as minocycline, may be associated with reduced gray matter loss in high-risk groups. However, it needs further studies (Miyaoka et al., 2008).

### 5.2 Limitations of interventions on pruning

Targeting synaptic pruning is a promising approach for preventing or mitigating neurodevelopmental disorders like schizophrenia. However, several important limitations and risks must be discussed.

First, synaptic pruning is a vital and evolutionarily conserved physiological process that refines neural circuits by eliminating weak or redundant synapses. This process optimizes cognitive and behavioral functions. Interfering with synaptic pruning, especially in a non-selective or poorly timed way, could disrupt critical brain maturation and lead to long-term issues such as cognitive overload, impaired learning, or autistic-like traits characterized by excessive synaptic connectivity (Tang et al., 2014; Gandal et al., 2018).

Second, current pharmacological interventions (summary in Table 2), including minocycline and kynurenine pathway modulators, operate on broad molecular pathways and have multiple effects. For instance, while minocycline reduces microglial activation, it may also impair neurogenesis or disrupt immune homeostasis (Miyaoka et al., 2008; Marchi et al., 2021). A practical approach probably should be time-limited (e.g., to adolescence or a prodromal stage), molecularly selective (e.g., only against hyperactive C4A), and reversible in action. An example is the use of sarcosine as an NMDA receptor modulator, which lowers glutamate levels and can reduce information noise in the hippocampus without permanently impairing plasticity (Tsai and Lin, 2010; Strzelecki et al., 2015). However, interfering with pruning may disrupt other processes of brain maturation and plasticity. Synaptic pruning is crucial for eliminating redundant connections, optimizing neural networks, facilitating memory formation, and promoting effective learning (Huttenlocher and Dabholkar, 1997). If microglial inhibition occurs too early or too strongly, it could prevent beneficial pruning and compromise synaptic plasticity, especially during adolescence, a time of active cortical remodeling. As previously noted, inhibiting the pruning in a non-selective manner can lead to network overload, adaptive deficits, or an excess of low-quality synapses (analogous to the phenotype observed in autism) (Tang et al., 2014; Gandal et al., 2018). Furthermore, suppressing microglial activity in certain models has been shown to impair emotional learning and memory formation on clinical level (Paolicelli et al., 2011).

**Table 2:** Summary of pharmacologic pruning-targeted strategies in schizophrenia.

Research focus	Mechanism	Clinical evidence	Key references
Tetracyclines (minocycline, doxycycline)	Anti-inflammatory, microglia-modulating; inhibits complement system and microglial phagocytosis	Reduced risk of psychosis in Swedish cohort (Sellgren et al., 2019); mixed results in BeneMin RCT (Deakin et al., 2018)	Miyaoka et al. (2008); Deakin et al. (2018); Sellgren et al. (2019); Li et al. (2021)
Complement system and C4	Excessive synaptic tagging via C1q/C3 and phagocytosis by microglia driven by C4A overexpression	Increased C4A expression linked to schizophrenia; indirect clinical targeting via microglial modulation	Schafer et al. (2012); Sekar et al. (2016); Sellgren et al. (2019); Chen et al. (2025)
Kynurenine pathway	KYNA inhibits NMDA; imbalance with QUIN promotes microglial activation and excitotoxicity	Altered KYNA: QUIN ratio in schizophrenia; early trials of KAT-II inhibitors (e.g., KYN-5356)	Schwarcz et al. (2012); Plitman et al. (2017); Pocivavsek et al. (2019); Marx et al. (2021)
Epigenetic modulation	Histone/DNA modifications (HDACs, DNMTs, LSD1) regulate pruning genes and microglial activity	Ongoing trial of LSD1 inhibitor vafidemstat; lifestyle epigenetic effects (e.g., exercise, diet)	Kaliman et al. (2014); Swathy and Banerjee (2017); Oryzon Genomics (2021); Park et al. (2022)
BDNF–NFκB Axis and neuroprotection	BDNF/TrkB promotes synaptic stability; NFκB mediates complement activation and cytokine signaling	BDNF upregulation via lifestyle and nutraceuticals (e.g., NAC, 7,8-DHF); preclinical validation	Minichiello (2009); Green et al. (2011); Hong et al. (2016b); Yu et al. (2018)
Cytokines and inflammation	IL-1β, IL-6, TNF-α drive microglial pruning via NFκB and complement induction	Elevated cytokines in ARMS/FEP; mixed results from trials with COX-2 and IL-6R inhibitors	Boulanger (2009); Missault et al. (2014); Mondelli et al. (2017); Khandaker et al. (2018)
Progranulin (GRN)	Protects synapses by suppressing microglial activation and C1q/C3 expression	GRN upregulation strategies under investigation (e.g., SORT1 inhibition, HDACi, CRISPR)	Petkau and Leavitt (2014); Lui et al. (2016); Paushter et al. (2018)
Gene regulators (SPI1, MEF2C, DISC1, RBFOX1)	Microglial/neuronal transcription factors regulate synaptic stability and pruning	Genetic association studies; animal models show pruning dysregulation and behavior changes	Kamiya et al. (2005); Harrington et al. (2016); O’Leary et al. (2022)
Other novel approaches (e.g., CBD, SCFA, neurosteroids)	Modulate microglia via CB2, TLR, gut-brain axis, and GABA-A related neurosteroids	Preclinical evidence; CANGLIA study with CBD ongoing	Mecha et al. (2016); Bossong (2017); Jolivel et al. (2021); Bäckström et al. (2024)

RCT, randomized controlled trial; C4A, complement component 4A; C1q/C3, complement proteins involved in synaptic tagging; KYNA, kynurenic acid; QUIN, quinolinic acid; NMDA, N-methyl-D-aspartate receptor; KAT-II, kynurenine aminotransferase II; HDAC, histone deacetylase; DNMT, DNA methyltransferase; LSD1, lysine-specific demethylase 1; NAC, N-acetylcysteine; 7,8-DHF, 7,8-dihydroxyflavone; NFκB, nuclear factor kappa-light-chain-enhancer of activated B cells; IL-1β, interleukin-1 beta; IL-6, interleukin-6; TNF-α, tumor necrosis factor alpha; ARMS, at-risk mental state; FEP, first-episode psychosis; COX-2, cyclooxygenase-2; IL-6R, interleukin-6 receptor; GRN, progranulin gene; SORT1, sortilin 1; SPI1, spleen focus-forming virus proviral integration oncogene; MEF2C, myocyte enhancer factor 2C; DISC1, disrupted-in-schizophrenia 1; RBFOX1, RNA-binding fox-1 homolog 1; CBD, cannabidiol; SCFA, short-chain fatty acids; CB2, cannabinoid receptor type 2; TLR, toll-like receptor; GABA-A, gamma-aminobutyric acid type A receptor.

Third, most clinical data on these interventions are limited or inconclusive. Some retrospective studies suggest that early exposure to tetracycline antibiotics might reduce the risk of psychosis (Sellgren et al., 2019). However, a prospective randomized trial, like BeneMin, failed to demonstrate clinical benefits of minocycline on negative symptoms or neuroimaging biomarkers in schizophrenia, although it may be too late for interventions, as main changes occurred earlier (Deakin et al., 2018). Likewise, therapies that modulate the complement system (e.g., C1q/C3 inhibitors) or the kynurenine pathway are still in preclinical or early clinical development, leaving their long-term effects on brain function unknown. From this perspective, it is worth emphasizing non-pharmacological options which, although ultimately less effective, should be assessed as safe (Table 3).

**Table 3:** Summary of non-pharmacologic pruning-targeted strategies potentially limiting excessive synaptic pruning in schizophrenia.

Intervention	Proposed mechanism related to pruning	Evidence	Key references
Lifestyle modification (stress reduction, sleep hygiene, exercise)	Reduces chronic stress and glucocorticoid-induced oxidative stress; promotes BDNF expression and synaptic plasticity	Clinical studies show exercise increases hippocampal volume and BDNF; sleep regulation linked to improved synaptic homeostasis	Pajonk et al. (2010); Wulff et al. (2010)
Omega-3 fatty acids supplementation	Anti-inflammatory, enhances BDNF, reduces microglial activation, protects dendritic spines	RCTs show reduced transition to psychosis in high-risk individuals	Amminger et al. (2010)
Dietary interventions (antioxidant-rich diet, sulforaphane from cruciferous vegetables)	Activation of Nrf2 pathway, increased glutathione, reduced oxidative damage to synapses	Small clinical studies: sulforaphane improved cognition in schizophrenia	Shiina et al. (2015); Hei et al. (2022)
Gut microbiota modulation (probiotics, prebiotics, FMT)	Influences microglial maturation and activity, regulates kynurenine pathway and inflammation	Preclinical evidence: microbiota transplantation improved synaptic plasticity; emerging early clinical data	Zheng et al. (2019); Cryan et al. (2020)
Cognitive remediation and social interventions	Stimulates activity-dependent synaptic strengthening, may counterbalance excessive elimination	Meta-analyses: improved cognition and functional connectivity in schizophrenia	Wykes et al. (2011)
Mindfulness, yoga, meditation	Stress reduction, decreased systemic inflammation, improved connectivity in default mode network	fMRI studies suggest improved regulation of neuroplasticity and inflammation	Cahn and Polich (2006)
Early detection and monitoring (“risk mapping”)	Identifying prodromal symptoms and family history allows preventive interventions before peak pruning	Family-history based high-risk cohorts show abnormal cortical thinning trajectories	Cannon et al. (2015)

BDNF, brain-derived neurotrophic factor; RCT, randomized controlled trial; Nrf2, nuclear factor erythroid 2–related factor 2; FMT, fecal microbiota transplantation; fMRI, functional magnetic resonance imaging.

Fourth, the heterogeneity of schizophrenia’s pathophysiology presents another challenge. Not all patients with schizophrenia exhibit excessive pruning or inflammatory markers. Consequently, interventions aimed at modulating pruning may only be beneficial for specific subgroups, such as those with elevated C4A expression or dysregulation in the kynurenine pathway, underscoring the need for stratified, biomarker-guided treatment approaches (Sekar et al., 2016; Orhan et al., 2023).

Finally, ethical concerns arise when considering preventive interventions during adolescence or prodromal stages, particularly in individuals who are asymptomatic. The risk–benefit ratio must be carefully evaluated, as altering fundamental neurodevelopmental mechanisms can have unpredictable long-term consequences. In summary, while modulating synaptic pruning is an intriguing hypothesis-driven target, it necessitates the use of highly selective, timing-sensitive, and reversible strategies. These strategies should ideally be combined with biomarker profiling and individualized risk assessment to avoid unintended neurodevelopmental trade-offs.

## 6 Conclusion

The hypothesis that excessive synaptic pruning during adolescence contributes to the pathogenesis of schizophrenia has opened new avenues for preventive strategies targeting neurodevelopmental processes. This review highlights a range of interventions, both pharmacological and molecular, that may modulate microglial activity and synaptic refinement in vulnerable individuals. These include tetracycline antibiotics (minocycline), modulation of the complement cascade (especially C4A), kynurenine pathway regulators (KYNA/QUIN balance), epigenetic therapies (LSD1, HDACs, lifestyle factors), neuroprotective agents such as BDNF and progranulin, and targeting specific transcription factors involved in pruning dynamics (e.g., MEF2C, PU.1). While these approaches demonstrate promise in preclinical and early clinical studies, several challenges remain. Pruning is a physiological process essential for healthy brain maturation and circuit optimization. Broad or untimely inhibition could lead to adverse developmental consequences, including cognitive overload or autism-like features. Furthermore, schizophrenia is a heterogeneous disorder, and hyperactive pruning may characterize only a subset of patients. Future interventions must therefore be precise and targeted at appropriate developmental windows, guided by biomarkers (e.g., C4A levels, kynurenine metabolites), and designed to preserve beneficial neuroplasticity. Ultimately, integrating mechanistic insight from molecular neuroscience with individualized clinical risk profiling may enable early, selective modulation of synaptic pruning. Such strategies hold the potential to alter disease trajectories and improve outcomes in schizophrenia, while minimizing unintended disruption of neurodevelopmental processes.

## References

[ref1] Aid T. Kazantseva A. Piirsoo M. Palm K. Timmusk T. (2007). Mouse and rat BDNF gene structure and expression revisited. J. Neurosci. Res. 85, 525–535. doi: 10.1002/jnr.21139, 17149751 PMC1878509

[ref2] Akbarian S. Huang H. S. (2009). Epigenetic regulation in human brain-focus on histone lysine methylation. Biol. Psychiatry 65, 198–203. doi: 10.1016/j.biopsych.2008.08.015, 18814864 PMC2637452

[ref3] Amado D. A. Rieders J. M. Diatta F. Hernandez-Con P. Singer A. Mak J. T. . (2019). AAV-mediated Progranulin delivery to a mouse model of progranulin deficiency causes T cell-mediated toxicity. Mol. Ther. 27, 465–478. doi: 10.1016/j.ymthe.2018.11.013, 30559071 PMC6369714

[ref4] Amminger G. P. Schäfer M. R. Papageorgiou K. Klier C. M. Cotton S. M. Harrigan S. M. . (2010). Long-chain ω-3 fatty acids for indicated prevention of psychotic disorders. Arch. Gen. Psychiatry 67, 146–154. doi: 10.1001/archgenpsychiatry.2009.192, 20124114

[ref5] Ancín I. Cabranes J. A. Vázquez-Álvarez B. Santos J. L. Sánchez-Morla E. Alaerts M. . (2013). NR4A2: effects of an orphan receptor on sustained attention in a schizophrenic population. Schizophr. Bull. 39, 555–563. doi: 10.1093/schbul/sbr176, 22294735 PMC3627752

[ref6] Angelucci F. Brenè S. Mathé A. A. (2005). BDNF in schizophrenia, depression and corresponding animal models. Mol. Psychiatry 10, 345–352. doi: 10.1038/sj.mp.4001637, 15655562

[ref7] Arrant A. E. Onyilo V. C. Unger D. E. Roberson E. D. (2018). Progranulin gene therapy improves lysosomal dysfunction and microglial pathology associated with frontotemporal dementia and neuronal ceroid lipofuscinosis. J. Neurosci. 38, 2341–2358. doi: 10.1523/JNEUROSCI.3081-17.2018, 29378861 PMC5830520

[ref8] Bäckström T. Doverskog M. Blackburn T. P. Scharschmidt B. F. Felipo V. (2024). Allopregnanolone and its antagonist modulate neuroinflammation and neurological impairment. Neurosci. Biobehav. Rev. 161:105668. doi: 10.1016/j.neubiorev.2024.105668, 38608826

[ref9] Balan I. Aurelian L. Schleicher R. Boero G. O’Buckley T. Morrow A. L. (2021). Neurosteroid allopregnanolone (3α,5α-THP) inhibits inflammatory signals induced by activated MyD88-dependent toll-like receptors. Transl. Psychiatry 11:145. doi: 10.1038/s41398-021-01266-1, 33637705 PMC7909379

[ref10] Battaglini M. Marino A. Montorsi M. Carmignani A. Ceccarelli M. C. Ciofani G. (2024). Nanomaterials as microglia modulators in the treatment of central nervous system disorders. Adv. Healthc. Mater. 13:4180. doi: 10.1002/adhm.202304180, 38112345

[ref11] Berk M. Copolov D. Dean O. Lu K. Jeavons S. Schapkaitz I. . (2008). N-acetyl cysteine as a glutathione precursor for schizophrenia-a double-blind, randomized, placebo-controlled trial. Biol. Psychiatry 64, 361–368. doi: 10.1016/j.biopsych.2008.03.004, 18436195

[ref12] Bernier D. Macintyre G. Bartha R. Hanstock C. C. McAllindon D. Cox D. . (2014). NPAS3 variants in schizophrenia: a neuroimaging study. BMC Med. Genet. 15:37. doi: 10.1186/1471-2350-15-37, 24674381 PMC3986669

[ref13] Bialas A. R. Stevens B. (2013). TGF-β signaling regulates neuronal C1q expression and developmental synaptic refinement. Nat. Neurosci. 16, 1773–1782. doi: 10.1038/nn.3560, 24162655 PMC3973738

[ref14] Birnbaum R. Weinberger D. R. (2017). Genetic insights into the neurodevelopmental origins of schizophrenia. Nat. Rev. Neurosci. 18, 727–740. doi: 10.1038/nrn.2017.125, 29070826

[ref15] Bitanihirwe B. K. Y. Woo T. U. W. (2011). Oxidative stress in schizophrenia: an integrated approach. Neurosci. Biobehav. Rev. 35, 878–893. doi: 10.1016/j.neubiorev.2010.10.008, 20974172 PMC3021756

[ref16] Bolton J. L. Short A. K. Othy S. Kooiker C. L. Shao M. Gunn B. G. . (2022). Early stress-induced impaired microglial pruning of excitatory synapses on immature CRH-expressing neurons provokes aberrant adult stress responses. Cell Rep. 38:110600. doi: 10.1016/j.celrep.2022.110600, 35354026 PMC9014810

[ref17] Bortz D. M. Wu H. Q. Schwarcz R. Bruno J. P. (2017). Oral administration of a specific kynurenic acid synthesis (KAT II) inhibitor attenuates evoked glutamate release in rat prefrontal cortex. Neuropharmacology 121, 69–78. doi: 10.1016/j.neuropharm.2017.04.023, 28419874 PMC5803791

[ref18] Bossong M. G. (2017). CANGLIA: Endocannabinoid control of microglia activation as a new therapeutic target in the treatment of schizophrenia.

[ref19] Boulanger L. M. (2009). Immune proteins in brain development and synaptic plasticity. Neuron 64, 93–109. doi: 10.1016/j.neuron.2009.09.001, 19840552

[ref20] Brundula V. Rewcastle N. B. Metz L. M. Bernard C. C. Yong V. W. (2002). Targeting leukocyte MMPs and transmigration minocycline as a potential therapy for multiple sclerosis. Brain 125, 1297–1308. doi: 10.1093/brain/awf133, 12023318

[ref21] Cahn B. R. Polich J. (2006). Meditation states and traits: EEG, ERP, and neuroimaging studies. Psychol. Bull. 132, 180–211. doi: 10.1037/0033-2909.132.2.180, 16536641

[ref22] Cakir B. Tanaka Y. Kiral F. R. Xiang Y. Dagliyan O. Wang J. . (2022). Expression of the transcription factor PU.1 induces the generation of microglia-like cells in human cortical organoids. Nat. Commun. 13:430. doi: 10.1038/s41467-022-28043-y, 35058453 PMC8776770

[ref23] Cannon T. D. Chung Y. He G. Sun D. Jacobson A. Van Erp T. G. M. . (2015). Progressive reduction in cortical thickness as psychosis develops: a multisite longitudinal neuroimaging study of youth at elevated clinical risk. Biol. Psychiatry 77:23. doi: 10.1016/j.biopsych.2014.05.023 PMC4264996 25034946

[ref24] Castro-Torres R. D. Olloquequi J. Parcerisas A. Ureña J. Ettcheto M. Beas-Zarate C. . (2024). JNK signaling and its impact on neural cell maturation and differentiation. Life Sci. 350:122750. doi: 10.1016/j.lfs.2024.122750, 38801982

[ref25] Cenik B. Sephton C. F. Cenik B. K. Herz J. Yu G. (2012). Progranulin: a proteolytically processed protein at the crossroads of inflammation and neurodegeneration. J. Biol. Chem. 287, 32298–32306. doi: 10.1074/jbc.R112.399170, 22859297 PMC3463300

[ref26] Chang C. Fonseca K. R. Li C. Horner W. Zawadzke L. E. Salafia M. A. . (2018). Quantitative translational analysis of brain kynurenic acid modulation via irreversible kynurenine aminotransferase II inhibition. Mol. Pharmacol. 94, 823–833. doi: 10.1124/mol.118.111625, 29853495

[ref27] Chen W. Tian Y. Gou M. Wang L. Tong J. Zhou Y. . (2024). Role of the immune-kynurenine pathway in treatment-resistant schizophrenia. Prog. Neuro-Psychopharmacol. Biol. Psychiatry 130:110926. doi: 10.1016/j.pnpbp.2023.110926, 38147973

[ref28] Chen Y. Zhao Z. Lin F. Wang L. Lin Z. Yue W. (2021). Associations between genotype and peripheral complement proteins in first-episode psychosis: evidences from C3 and C4. Front. Genet. 12:647246. doi: 10.3389/fgene.2021.647246, 34306006 PMC8301372

[ref29] Chen Z.-P. Zhao X. Wang S. Cai R. Liu Q. Ye H. . (2025). GABA-dependent microglial elimination of inhibitory synapses underlies neuronal hyperexcitability in epilepsy. Nat. Neurosci. 28, 1404–1417. doi: 10.1038/s41593-025-01979-2, 40425792

[ref30] Choi G. B. Yim Y. S. Wong H. Kim S. Kim H. Kim S. V. . (2016). The maternal interleukin-17a pathway in mice promotes autism-like phenotypes in offspring. Science 351, 933–939. doi: 10.1126/science.aad0314, 26822608 PMC4782964

[ref31] Christopher M. A. Kyle S. M. Katz D. J. (2017). Neuroepigenetic mechanisms in disease. Epigenetics Chromatin 10:47. doi: 10.1186/s13072-017-0150-4, 29037228 PMC5644115

[ref32] Chunchai T. Thunapong W. Yasom S. Wanchai K. Eaimworawuthikul S. Metzler G. . (2018). Decreased microglial activation through gut-brain Axis by prebiotics, probiotics, or synbiotics effectively restored cognitive function in obese-insulin resistant rats. J. Neuroinflammation 15:11. doi: 10.1186/s12974-018-1055-2, 29316965 PMC5761137

[ref33] Chung W. S. Clarke L. E. Wang G. X. Stafford B. K. Sher A. Chakraborty C. . (2013). Astrocytes mediate synapse elimination through MEGF10 and MERTK pathways. Nature 504, 394–400. doi: 10.1038/nature12776, 24270812 PMC3969024

[ref34] Chung Y. Dienel S. J. Belch M. J. Fish K. N. Ermentrout G. B. Lewis D. A. . (2024). Altered Rbfox1-Vamp1 pathway and prefrontal cortical dysfunction in schizophrenia. Mol. Psychiatry 29, 1382–1391. doi: 10.1038/s41380-024-02417-8, 38273110 PMC11273323

[ref35] Conus P. Seidman L. J. Fournier M. Xin L. Cleusix M. Baumann P. S. . (2018). N-acetylcysteine in a double-blind randomized placebo-controlled trial: toward biomarker-guided treatment in early psychosis. Schizophr. Bull. 44, 317–327. doi: 10.1093/schbul/sbx093, 29462456 PMC5815074

[ref36] Corley S. M. Tsai S. Y. Wilkins M. R. Weickert C. S. (2016). Transcriptomic analysis shows decreased cortical expression of nr4a1, nr4a2 and rxrb in schizophrenia and provides evidence for nuclear receptor dysregulation. PLoS One 11:e166944. doi: 10.1371/journal.pone.0166944, 27992436 PMC5161508

[ref37] Cryan J. F. O’Riordan K. J. Sandhu K. Peterson V. Dinan T. G. (2020). The gut microbiome in neurological disorders. Lancet Neurol. 19, 179–194. doi: 10.1016/S1474-4422(19)30356-4 31753762

[ref38] D’Amato A. Di Cesare Mannelli L. Lucarini E. Man A. L. Le Gall G. Branca J. J. V. . (2020). Faecal microbiota transplant from aged donor mice affects spatial learning and memory via modulating hippocampal synaptic plasticity- and neurotransmission-related proteins in young recipients. Microbiome 8:140. doi: 10.1186/s40168-020-00914-w, 33004079 PMC7532115

[ref39] Dahoun T. Trossbach S. V. Brandon N. J. Korth C. Howes O. D. (2017). The impact of disrupted-in-schizophrenia 1 (DISC1) on the dopaminergic system: a systematic review. Transl. Psychiatry 7:e1015. doi: 10.1038/tp.2016.282, 28140405 PMC5299392

[ref40] de Almeida V. Martins-de-Souza D. (2018). Cannabinoids and glial cells: possible mechanism to understand schizophrenia. Eur. Arch. Psychiatry Clin. Neurosci. 268, 727–737. doi: 10.1007/s00406-018-0874-6, 29392440

[ref41] de Bie J. Lim C. K. Guillemin G. J. (2016). Kynurenines, gender and Neuroinflammation; showcase schizophrenia. Neurotox. Res. 30, 285–294. doi: 10.1007/s12640-016-9641-5, 27342132

[ref42] Deakin B. Suckling J. Barnes T. R. E. Byrne K. Chaudhry I. B. Dazzan P. . (2018). The benefit of minocycline on negative symptoms of schizophrenia in patients with recent-onset psychosis (BeneMin): a randomised, double-blind, placebo-controlled trial. Lancet Psychiatry 5, 885–894. doi: 10.1016/S2215-0366(18)30345-6, 30322824 PMC6206257

[ref43] Dickerson F. Origoni A. Katsafanas E. Squire A. Newman T. Fahey J. . (2021). Randomized controlled trial of an adjunctive sulforaphane nutraceutical in schizophrenia. Schizophr. Res. 231, 142–144. doi: 10.1016/j.schres.2021.03.018, 33839372

[ref44] Do K. Q. Cabungcal J. H. Frank A. Steullet P. Cuenod M. (2009). Redox dysregulation, neurodevelopment, and schizophrenia. Curr. Opin. Neurobiol. 19, 220–230. doi: 10.1016/j.conb.2009.05.001, 19481443

[ref45] Erhardt S. Schwieler L. Imbeault S. Engberg G. (2007). The kynurenine pathway in schizophrenia and bipolar disorder. Neuropharmacology 53, 601–611. doi: 10.1016/j.neuropharm.2007.07.012 27245499

[ref46] Feinberg I. (1982). Schizophrenia: caused by a fault in programmed synaptic elimination during adolescence? J. Psychiatr. Res. 17, 319–334. doi: 10.1016/0022-3956(82)90038-3, 7187776

[ref47] Fernandes B. S. Steiner J. Berk M. Molendijk M. L. Gonzalez-Pinto A. Turck C. W. . (2015). Peripheral brain-derived neurotrophic factor in schizophrenia and the role of antipsychotics: meta-analysis and implications. Mol. Psychiatry 20, 1108–1119. doi: 10.1038/mp.2014.117, 25266124

[ref48] Ferranti A. S. Foster D. J. (2022). Cannabinoid type-2 receptors: an emerging target for regulating schizophrenia-relevant brain circuits. Front. Neurosci. 16:925792. doi: 10.3389/fnins.2022.925792, 36033626 PMC9403189

[ref49] Fu X. Zhang G. Liu Y. Zhang L. Zhang F. Zhou C. (2020). Altered expression of the DISC1 gene in peripheral blood of patients with schizophrenia. BMC Med. Genet. 21:194. doi: 10.1186/s12881-020-01132-9, 33008326 PMC7532617

[ref50] Gandal M. J. Haney J. R. Parikshak N. N. Leppa V. Ramaswami G. Hartl C. . (2018). Shared molecular neuropathology across major psychiatric disorders parallels polygenic overlap. Science 359, 693–697. doi: 10.1126/science.aad6469, 32015716 PMC6996074

[ref51] Garay P. A. Hsiao E. Y. Patterson P. H. McAllister A. K. (2013). Maternal immune activation causes age- and region-specific changes in brain cytokines in offspring throughout development. Brain Behav. Immun. 31, 54–68. doi: 10.1016/j.bbi.2012.07.008, 22841693 PMC3529133

[ref52] Garrido-Mesa N. Zarzuelo A. Gálvez J. (2013). Minocycline: far beyond an antibiotic. Br. J. Pharmacol. 169, 337–352. doi: 10.1111/bph.12139, 23441623 PMC3651660

[ref53] Gehman L. T. Stoilov P. Maguire J. Damianov A. Lin C. H. Shiue L. . (2011). The splicing regulator Rbfox1 (A2BP1) controls neuronal excitation in the mammalian brain. Nat. Genet. 43, 706–711. doi: 10.1038/ng.841, 21623373 PMC3125461

[ref54] Germann M. Brederoo S. G. Sommer I. E. C. (2021). Abnormal synaptic pruning during adolescence underlying the development of psychotic disorders. Curr. Opin. Psychiatry 34, 222–227. doi: 10.1097/YCO.0000000000000696, 33560023 PMC8048735

[ref55] Glantz L. A. Lewis D. A. (2000). Decreased dendritic spine density on prefrontal cortical pyramidal neurons in schizophrenia. Arch. Gen. Psychiatry 57:65. doi: 10.1001/archpsyc.57.1.65, 10632234

[ref56] Glausier J. R. Lewis D. A. (2013). Dendritic spine pathology in schizophrenia. Neuroscience 251, 90–107. doi: 10.1016/j.neuroscience.2012.04.044, 22546337 PMC3413758

[ref57] Goker M. Aytac H. M. Guclu O. (2023). Evaluation of serum complement levels and factors affecting treatment resistance in patients with schizophrenia. Psychiatry Clin. Psychopharmacol. 33, 84–93. doi: 10.5152/pcp.2023.22580, 38765923 PMC11082620

[ref58] Gómez-Pinilla F. (2008). Brain foods: the effects of nutrients on brain function. Nat. Rev. Neurosci. 9, 568–578. doi: 10.1038/nrn2421, 18568016 PMC2805706

[ref59] Green M. J. Matheson S. L. Shepherd A. Weickert C. S. Carr V. J. (2011). Brain-derived neurotrophic factor levels in schizophrenia: a systematic review with meta-analysis. Mol. Psychiatry 16, 960–972. doi: 10.1038/mp.2010.88, 20733577

[ref60] Guan J. S. Haggarty S. J. Giacometti E. Dannenberg J. H. Joseph N. Gao J. . (2009). HDAC2 negatively regulates memory formation and synaptic plasticity. Nature 459, 55–60. doi: 10.1038/nature07925, 19424149 PMC3498958

[ref61] Hao W. Ma Q. Wang L. Yuan N. Gan H. He L. . (2024). Gut dysbiosis induces the development of depression-like behavior through abnormal synapse pruning in microglia-mediated by complement C3. Microbiome 12:34. doi: 10.1186/s40168-024-01756-6, 38378622 PMC10877840

[ref62] Haroon E. Miller A. H. Sanacora G. (2017). Inflammation, glutamate, and glia: a trio of trouble in mood disorders. Neuropsychopharmacology 42, 193–215. doi: 10.1038/npp.2016.199, 27629368 PMC5143501

[ref63] Harrington A. J. Raissi A. Rajkovich K. Berto S. Kumar J. Molinaro G. . (2016). MEF2C regulates cortical inhibitory and excitatory synapses and behaviors relevant to neurodevelopmental disorders. eLife 5:e20059. doi: 10.7554/eLife.20059, 27779093 PMC5094851

[ref64] Hei G. Smith R. C. Li R. Ou J. Song X. Zheng Y. . (2022). Sulforaphane effects on cognition and symptoms in first and early episode schizophrenia: a randomized double-blind trial. Schizophr. Bull. Open 3:sgac024. doi: 10.1093/schizbullopen/sgac024, 39144775 PMC11205988

[ref65] Henry C. J. Huang Y. Wynne A. M. Godbout J. P. (2009). Peripheral lipopolysaccharide (LPS) challenge promotes microglial hyperactivity in aged mice that is associated with exaggerated induction of both pro-inflammatory IL-1β and anti-inflammatory IL-10 cytokines. Brain Behav. Immun. 23, 309–317. doi: 10.1016/j.bbi.2008.09.002, 18814846 PMC2692986

[ref66] Hikida T. Jaaro-Peled H. Seshadri S. Oishi K. Hookway C. Kong S. . (2007). Dominant-negative DISC1 transgenic mice display schizophrenia-associated phenotypes detected by measures translatable to humans. Proc. Natl. Acad. Sci. USA 104, 14501–14506. doi: 10.1073/pnas.0704774104, 17675407 PMC1964873

[ref67] Holler C. J. Taylor G. McEachin Z. T. Deng Q. Watkins W. J. Hudson K. . (2016). Trehalose upregulates progranulin expression in human and mouse models of GRN haploinsufficiency: a novel therapeutic lead to treat frontotemporal dementia. Mol. Neurodegener. 11:46. doi: 10.1186/s13024-016-0114-3, 27341800 PMC4919863

[ref68] Hölzel B. K. Carmody J. Vangel M. Congleton C. Yerramsetti S. M. Gard T. . (2011). Mindfulness practice leads to increases in regional brain gray matter density. Psychiatry Res. Neuroimaging 191, 36–43. doi: 10.1016/j.pscychresns.2010.08.006, 21071182 PMC3004979

[ref69] Hong S. Beja-Glasser V. F. Nfonoyim B. M. Frouin A. Li S. Ramakrishnan S. . (2016a). Complement and microglia mediate early synapse loss in Alzheimer mouse models. Science 352, 712–716. doi: 10.1126/science.aad8373, 27033548 PMC5094372

[ref70] Hong S. Dissing-Olesen L. Stevens B. (2016b). New insights on the role of microglia in synaptic pruning in health and disease. Curr. Opin. Neurobiol. 36, 128–134. doi: 10.1016/j.conb.2015.12.004, 26745839 PMC5479435

[ref71] Hu F. Padukkavidana T. Vægter C. B. Brady O. A. Zheng Y. Mackenzie I. R. . (2010). Sortilin-mediated endocytosis determines levels of the frontotemporal dementia protein, progranulin. Neuron 68, 654–667. doi: 10.1016/j.neuron.2010.09.034, 21092856 PMC2990962

[ref72] Huttenlocher P. R. Dabholkar A. S. (1997). Regional differences in synaptogenesis in human cerebral cortex. J. Comp. Neurol. 387, 167–178. doi: 10.1002/(SICI)1096-9861(19971020)387:2<167::AID-CNE1>3.0.CO;2-Z, 9336221

[ref73] Inam M. E. Fernandes B. S. Salagre E. Grande I. Vieta E. Quevedo J. . (2023). The kynurenine pathway in major depressive disorder, bipolar disorder, and schizophrenia: a systematic review and meta-analysis of cerebrospinal fluid studies. Braz. J. Psychiatry 45, 343–355. doi: 10.47626/1516-4446-2022-2973, 37127280 PMC10668321

[ref74] Inta D. Lang U. E. Borgwardt S. Meyer-Lindenberg A. Gass P. (2017). Microglia activation and schizophrenia: lessons from the effects of minocycline on postnatal neurogenesis, neuronal survival and synaptic pruning. Schizophr. Bull. 43, 493–496. doi: 10.1093/schbul/sbw088, 27352782 PMC5464012

[ref75] Inta I. Vogt M. A. Vogel A. S. Bettendorf M. Gass P. Inta D. (2016). Minocycline exacerbates apoptotic neurodegeneration induced by the NMDA receptor antagonist MK-801 in the early postnatal mouse brain. Eur. Arch. Psychiatry Clin. Neurosci. 266, 673–677. doi: 10.1007/s00406-015-0649-2, 26482736

[ref76] Jebelli J. Hooper C. Pocock J. M. (2014). Microglial p53 activation is detrimental to neuronal synapses during activation-induced inflammation: implications for neurodegeneration. Neurosci. Lett. 583, 92–97. doi: 10.1016/j.neulet.2014.08.049, 25204787

[ref77] Johnstone A. Mobley W. (2020). Local TrkB signaling: themes in development and neural plasticity. Cell Tissue Res. 382, 101–111. doi: 10.1007/s00441-020-03278-7, 32936344

[ref78] Jolivel V. Brun S. Binamé F. Benyounes J. Taleb O. Bagnard D. . (2021). Microglial cell morphology and phagocytic activity are critically regulated by the neurosteroid allopregnanolone: a possible role in neuroprotection. Cells 10:698. doi: 10.3390/cells10030698, 33801063 PMC8004004

[ref79] Ju S. Shin Y. Han S. Kwon J. Choi T. G. Kang I. . (2023). The gut–brain axis in schizophrenia: the implications of the gut microbiome and SCFA production. Nutrients 15:4391. doi: 10.3390/nu15204391, 37892465 PMC10610543

[ref80] Kaliman P. Álvarez-López M. J. Cosín-Tomás M. Rosenkranz M. A. Lutz A. Davidson R. J. (2014). Rapid changes in histone deacetylases and inflammatory gene expression in expert meditators. Psychoneuroendocrinology 40, 96–107. doi: 10.1016/j.psyneuen.2013.11.004, 24485481 PMC4039194

[ref81] Kaltschmidt B. Kaltschmidt C. (2009). NF-kappaB in the nervous system. Cold Spring Harb. Perspect. Biol. 1:1271. doi: 10.1101/cshperspect.a001271, 20066105 PMC2773634

[ref82] Kamiya A. Kubo K. I. Tomoda T. Takaki M. Youn R. Ozeki Y. . (2005). A schizophrenia-associated mutation of DISC1 perturbs cerebral cortex development. Nat. Cell Biol. 7, 1167–1178. doi: 10.1038/ncb1328, 16299498

[ref83] Khandaker G. M. Oltean B. P. Kaser M. Dibben C. R. M. Ramana R. Jadon D. R. . (2018). Protocol for the insight study: a randomised controlled trial of singledose tocilizumab in patients with depression and low-grade inflammation. BMJ Open 8:e025333. doi: 10.1136/bmjopen-2018-025333, 30244217 PMC6157523

[ref84] Kim H. S. Suh Y. H. (2009). Minocycline and neurodegenerative diseases. Behav. Brain Res. 196, 168–179. doi: 10.1016/j.bbr.2008.09.040, 18977395

[ref85] Knuesel I. Chicha L. Britschgi M. Schobel S. A. Bodmer M. Hellings J. A. . (2014). Maternal immune activation and abnormal brain development across CNS disorders. Nat. Rev. Neurol. 10, 643–660. doi: 10.1038/nrneurol.2014.187, 25311587

[ref86] Kolluri N. Sun Z. Sampson A. R. Lewis D. A. (2005). Lamina-specific reductions in dendritic spine density in the prefrontal cortex of subjects with schizophrenia. Am. J. Psychiatry 162, 1200–1202. doi: 10.1176/appi.ajp.162.6.1200, 15930070

[ref9001] Konopaske G. T. Lange N. Coyle J. T. Benes F. M. (2014). Prefrontal cortical dendritic spine pathology in schizophrenia and bipolar disorder. JAMA Psychiatry. 71, 1323–1331. doi: 10.1001/jamapsychiatry.2014.1582 PMC5510541 25271938

[ref87] Kouzarides T. (2007). Chromatin modifications and their function. Cell 128, 693–705. doi: 10.1016/j.cell.2007.02.005, 17320507

[ref88] Kozak R. Campbell B. M. Strick C. A. Horner W. Hoffmann W. E. Kiss T. . (2014). Reduction of brain kynurenic acid improves cognitive function. J. Neurosci. 34, 10592–10602. doi: 10.1523/JNEUROSCI.1107-14.2014, 25100593 PMC6802596

[ref89] Kynexis Therapeutics (2024). Kynexis announces positive topline results from phase 1 study of KYN-5356, a potential treatment for cognitive impairment associated with schizophrenia.

[ref90] Laan W. Grobbee D. E. Selten J. P. Heijnen C. J. Kahn R. S. Burger H. (2010). Adjuvant aspirin therapy reduces symptoms of schizophrenia spectrum disorders: results from a randomized, double-blind, placebo-controlled trial. J. Clin. Psychiatry 71, 520–527. doi: 10.4088/JCP.09m05117yel, 20492850

[ref91] Laskaris L. Zalesky A. Weickert C. S. Di Biase M. A. Chana G. Baune B. T. . (2019). Investigation of peripheral complement factors across stages of psychosis. Schizophr. Res. 204, 30–37. doi: 10.1016/j.schres.2018.11.035, 30527272

[ref92] Li Q. Cheng Z. Zhou L. Darmanis S. Zhang Y. Pan J. (2021). Minocycline attenuates complement C3 activation and microglial phagocytosis via the C1q/C3-CR3 axis in intracerebral hemorrhage. Neuropharmacology 186:108474. doi: 10.1016/j.neuropharm.2020.108474

[ref93] Linderholm K. R. Skogh E. Olsson S. K. Dahl M. L. Holtze M. Engberg G. . (2012). Increased levels of kynurenine and kynurenic acid in the CSF of patients with schizophrenia. Schizophr. Bull. 38, 426–432. doi: 10.1093/schbul/sbq086, 20729465 PMC3329991

[ref94] Liu B. Zhang Y. Yang Z. Liu M. Zhang C. Zhao Y. . (2021). ω-3 DPA protected neurons from Neuroinflammation by balancing microglia M1/M2 polarizations through inhibiting NF-κB/MAPK p38 signaling and activating neuron-BDNF-PI3K/AKT pathways. Mar. Drugs 19:587. doi: 10.3390/md19110587, 34822458 PMC8619469

[ref95] Lui H. Zhang J. Makinson S. R. Cahill M. K. Kelley K. W. Huang H. Y. . (2016). Progranulin deficiency promotes circuit-specific synaptic pruning by microglia via complement activation. Cell 165, 921–935. doi: 10.1016/j.cell.2016.04.001, 27114033 PMC4860138

[ref96] Madore C. Leyrolle Q. Morel L. Rossitto M. Greenhalgh A. D. Delpech J. C. . (2020). Essential omega-3 fatty acids tune microglial phagocytosis of synaptic elements in the mouse developing brain. Nat. Commun. 11:6133. doi: 10.1038/s41467-020-19861-z, 33257673 PMC7704669

[ref97] Makinodan M. Rosen K. M. Ito S. Corfas G. (2012). A critical period for social experience-dependent oligodendrocyte maturation and myelination. Science 337, 1357–1360. doi: 10.1126/science.1220845, 22984073 PMC4165613

[ref98] Mallya A. P. Wang H. D. Lee H. N. R. Deutch A. Y. (2019). Microglial pruning of synapses in the prefrontal cortex during adolescence. Cereb. Cortex 29, 1634–1643. doi: 10.1093/cercor/bhy061, 29668872 PMC6418387

[ref99] Marchi M. Galli G. Magarini F. M. Mattei G. Galeazzi G. M. (2021). Sarcosine as an add-on treatment to antipsychotic medication for people with schizophrenia: a systematic review and meta-analysis of randomized controlled trials. Expert Opin. Drug Metab. Toxicol. 17, 483–493. doi: 10.1080/17425255.2021.1885648, 33538213

[ref100] Martens L. H. Zhang J. Barmada S. J. Zhou P. Kamiya S. Sun B. . (2012). Progranulin deficiency promotes neuroinflammation and neuron loss following toxin-induced injury. J. Clin. Invest. 122, 3955–3959. doi: 10.1172/JCI63113, 23041626 PMC3484443

[ref101] Marx W. McGuinness A. J. Rocks T. Ruusunen A. Cleminson J. Walker A. J. . (2021). The kynurenine pathway in major depressive disorder, bipolar disorder, and schizophrenia: a meta-analysis of 101 studies. Mol. Psychiatry 26, 4158–4178. doi: 10.1038/s41380-020-00951-9, 33230205

[ref102] McEwen B. S. Morrison J. H. (2013). The brain on stress: vulnerability and plasticity of the prefrontal cortex over the life course. Neuron 79, 16–29. doi: 10.1016/j.neuron.2013.06.028, 23849196 PMC3753223

[ref103] Mecha M. Carrillo-Salinas F. J. Feliú A. Mestre L. Guaza C. (2016). Microglia activation states and cannabinoid system: therapeutic implications. Pharmacol. Ther. 166, 40–55. doi: 10.1016/j.pharmthera.2016.06.011, 27373505

[ref104] Minichiello L. (2009). TrkB signalling pathways in LTP and learning. Nat. Rev. Neurosci. 10, 850–860. doi: 10.1038/nrn2738, 19927149

[ref105] Missault S. Van den Eynde K. Vanden Berghe W. Fransen E. Weeren A. Timmermans J. P. . (2014). The risk for behavioural deficits is determined by the maternal immune response to prenatal immune challenge in a neurodevelopmental model. Brain Behav. Immun. 42, 138–146. doi: 10.1016/j.bbi.2014.06.013, 24973728

[ref106] Miyaoka T. Yasukawa R. Yasuda H. Hayashida M. Inagaki T. Horiguchi J. (2008). Minocycline as adjunctive therapy for schizophrenia: an open-label study. Clin. Neuropharmacol. 31, 287–292. doi: 10.1097/WNF.0b013e3181593d45, 18836347

[ref107] Mohd Asyraf A. J. Nour El Huda A. R. Hanisah M. N. Norsidah K. Z. Norlelawati A. T. (2022). Relationship of selective complement markers with schizophrenia. J. Neuroimmunol. 363:577793. doi: 10.1016/j.jneuroim.2021.577793, 34990981

[ref108] Mondelli V. Cattaneo A. Murri M. B. Papadopoulos A. S. Aitchison K. J. (2011). Stress and inflammation reduce BDNF expression in first- episode psychosis: a pathway to smaller hippocampal volume. J. Clin. Psychiatry 72, 1677–1684. doi: 10.4088/JCP.10m06745, 21672499 PMC4082665

[ref109] Mondelli V. Vernon A. C. Turkheimer F. Dazzan P. Pariante C. M. (2017). Brain microglia in psychiatric disorders. Lancet Psychiatry 4, 563–572. doi: 10.1016/S2215-0366(17)30101-3, 28454915

[ref110] Morini R. Bizzotto M. Perrucci F. Filipello F. Matteoli M. (2021). Strategies and tools for studying microglial-mediated synapse elimination and refinement. Front. Immunol. 12:640937. doi: 10.3389/fimmu.2021.640937, 33708226 PMC7940197

[ref111] Müller N. Krause D. Dehning S. Musil R. Schennach-Wolff R. Obermeier M. . (2010). Celecoxib treatment in an early stage of schizophrenia: results of a randomized, double-blind, placebo-controlled trial of celecoxib augmentation of amisulpride treatment. Schizophr. Res. 121, 118–124. doi: 10.1016/j.schres.2010.04.015, 20570110

[ref112] Mullier E. Roine T. Griffa A. Xin L. Baumann P. S. Klauser P. . (2019). N-acetyl-cysteine supplementation improves functional connectivity within the cingulate cortex in early psychosis: a pilot study. Int. J. Neuropsychopharmacol. 22, 478–487. doi: 10.1093/ijnp/pyz022, 31283822 PMC6672595

[ref113] Mushtaq R. Khan M. M. Fatima A. (2015). Clinical efficacy of minocycline and/or omega-3 fatty acids in individuals with at-risk mental State (ARMS): a randomized controlled trial protocol.

[ref114] Niwa M. Jaaro-Peled H. Tankou S. Seshadri S. Hikida T. Matsumoto Y. . (2013). Adolescent stress-induced epigenetic control of dopaminergic neurons via glucocorticoids. Science 339, 335–339. doi: 10.1126/science.1226931, 23329051 PMC3617477

[ref115] Norkett R. Modi S. Birsa N. Atkin T. A. Ivankovic D. Pathania M. . (2016). DISC1-dependent regulation of mitochondrial dynamics controls the morphogenesis of complex neuronal dendrites. J. Biol. Chem. 291, 613–629. doi: 10.1074/jbc.M115.699447, 26553875 PMC4705382

[ref116] O’Leary A. Fernàndez-Castillo N. Gan G. Yang Y. Yotova A. Y. Kranz T. M. . (2022). Behavioural and functional evidence revealing the role of RBFOX1 variation in multiple psychiatric disorders and traits. Mol. Psychiatry 27, 4464–4473. doi: 10.1038/s41380-022-01722-4, 35948661 PMC9734045

[ref117] Orhan F. Malwade S. Schwieler L. Tiihonen J. Koistinahu J. Erhardt S. . (2023). Kynurenic acid promotes activity-dependent synaptic pruning and associates with genetic risk variance for schizophrenia. Neurosci. Appl. 2:3551. doi: 10.1016/j.nsa.2023.103551

[ref118] Oryzon Genomics (2021). Oryzon presents positive results from Phase IIa trial with vafidemstat in schizophrenia (EVOLUTION trial). Available online at: https://www.oryzon.com/sites/default/files/events-presentations/oryzon_evotuionresults_june2021.pdf (Accessed April 17, 2025).

[ref119] Pajonk F. G. Wobrock T. Gruber O. Scherk H. Berner D. Kaizl I. . (2010). Hippocampal plasticity in response to exercise in schizophrenia. Arch. Gen. Psychiatry 67, 133–143. doi: 10.1001/archgenpsychiatry.2009.193, 20124113

[ref120] Palzur E. Edelman D. Sakas R. Soustiel J. F. (2021). Etifoxine restores mitochondrial oxidative phosphorylation and improves cognitive recovery following traumatic brain injury. Int. J. Mol. Sci. 22:2881. doi: 10.3390/ijms222312881, 34884686 PMC8657969

[ref121] Paolicelli R. C. Bolasco G. Pagani F. Maggi L. Scianni M. Panzanelli P. . (2011). Synaptic pruning by microglia is necessary for normal brain development. Science 333, 1456–1458. doi: 10.1126/science.1202529, 21778362

[ref122] Pardo C. A. Vargas D. L. Zimmerman A. W. (2005). Immunity, neuroglia and neuroinflammation in autism. Int. Rev. Psychiatry 17, 485–495. doi: 10.1080/02646830500381930, 16401547

[ref123] Park J. Lee K. Kim K. Yi S. J. (2022). The role of histone modifications: from neurodevelopment to neurodiseases. Signal Transduct. Target. Ther. 7:217. doi: 10.1038/s41392-022-01078-9, 35794091 PMC9259618

[ref124] Paushter D. H. Du H. Feng T. Hu F. (2018). The lysosomal function of progranulin, a guardian against neurodegeneration. Acta Neuropathol. 136, 1–17. doi: 10.1007/s00401-018-1861-8, 29744576 PMC6117207

[ref125] Pedraz-Petrozzi B. Elyamany O. Rummel C. Mulert C. (2020). Effects of inflammation on the kynurenine pathway in schizophrenia - a systematic review. J. Neuroinflammation 17:56. doi: 10.1186/s12974-020-1721-z, 32061259 PMC7023707

[ref126] Perry V. H. Holmes C. (2014). Microglial priming in neurodegenerative disease. Nat. Rev. Neurol. 10, 217–224. doi: 10.1038/nrneurol.2014.38, 24638131

[ref127] Petkau T. L. Leavitt B. R. (2014). Progranulin in neurodegenerative disease. Trends Neurosci. 37, 388–398. doi: 10.1016/j.tins.2014.04.003, 24800652

[ref128] Pettus E. H. Wright D. W. Stein D. G. Hoffman S. W. (2005). Progesterone treatment inhibits the inflammatory agents that accompany traumatic brain injury. Brain Res. 1049, 112–119. doi: 10.1016/j.brainres.2005.05.004, 15932748

[ref129] Plane J. M. Shen Y. Pleasure D. E. Deng W. (2010). Prospects for minocycline neuroprotection. Arch. Neurol. 67, 1442–1448. doi: 10.1001/archneurol.2010.191, 20697034 PMC3127230

[ref130] Plitman E. Iwata Y. Caravaggio F. Nakajima S. Chung J. K. Gerretsen P. . (2017). Kynurenic acid in schizophrenia: a systematic review and Meta-analysis. Schizophr. Bull. 43, 764–777. doi: 10.1093/schbul/sbw221, 28187219 PMC5472151

[ref131] Pocivavsek A. Elmer G. I. Schwarcz R. (2019). Inhibition of kynurenine aminotransferase II attenuates hippocampus-dependent memory deficit in adult rats treated prenatally with kynurenine. Hippocampus 29, 73–77. doi: 10.1002/hipo.23040, 30311334 PMC6367926

[ref132] Popovic D. Schmitt A. Kaurani L. Senner F. Papiol S. Malchow B. . (2019). Childhood trauma in schizophrenia: current findings and research perspectives. Front. Neurosci. 13:274. doi: 10.3389/fnins.2019.00274, 30983960 PMC6448042

[ref133] Raitano S. Ordovàs L. De Muynck L. Guo W. Espuny-Camacho I. Geraerts M. . (2015). Restoration of progranulin expression rescues cortical neuron generation in an induced pluripotent stem cell model of frontotemporal dementia. Stem Cell Rep. 4, 16–24. doi: 10.1016/j.stemcr.2014.12.001, 25556567 PMC4297877

[ref134] Rapoport J. L. Giedd J. N. Gogtay N. (2012). Neurodevelopmental model of schizophrenia: update 2012. Mol. Psychiatry 17, 1228–1238. doi: 10.1038/mp.2012.23, 22488257 PMC3504171

[ref135] Ripke S. Neale B. M. Corvin A. Walters J. T. R. Farh K. H. Holmans P. A. . (2014). Biological insights from 108 schizophrenia-associated genetic loci. Nature 511:16595. doi: 10.1038/nature13595 PMC4112379 25056061

[ref136] Rodrigues da Silva N. Gobbo D. Gomes F. V. Scheller A. Kirchhoff F. Del Bel E. . (2025). Cannabidiol reverses microglia activation and deficits of parvalbumin interneurons and their perineuronal nets in a MK-801-induced mouse model of schizophrenia. Brain Res. 1863:149772. doi: 10.1016/j.brainres.2025.149772, 40484109

[ref137] Rojas P. Joodmardi E. Hong Y. Perlmann T. Ögren S. O. (2007). Adult mice with reduced Nurr1 expression: an animal model for schizophrenia. Mol. Psychiatry 12, 756–766. doi: 10.1038/sj.mp.4001993, 17457314

[ref138] Rosato M. Stringer S. Gebuis T. Paliukhovich I. Li K. W. Posthuma D. . (2021). Combined cellomics and proteomics analysis reveals shared neuronal morphology and molecular pathway phenotypes for multiple schizophrenia risk genes. Mol. Psychiatry 26, 784–799. doi: 10.1038/s41380-019-0436-y, 31142819 PMC7910218

[ref139] Roux L. Stark E. Sjulson L. Buzsáki G. (2023). In vivo optogenetic identification and manipulation of GABAergic interneuron subtypes. arXiv, 24440414 10.1016/j.conb.2013.12.013 PMC4024355

[ref140] Rupprecht R. Papadopoulos V. Rammes G. Baghai T. C. Fan J. Akula N. . (2010). Translocator protein (18 kDa) (TSPO) as a therapeutic target for neurological and psychiatric disorders. Nat. Rev. Drug Discov. 9, 971–988. doi: 10.1038/nrd3295, 21119734

[ref141] Rupprecht R. Pradhan A. K. Kufner M. Brunner L. M. Nothdurfter C. Wein S. . (2023). Neurosteroids and translocator protein 18 kDa (TSPO) in depression: implications for synaptic plasticity, cognition, and treatment options. Eur. Arch. Psychiatry Clin. Neurosci. 273, 1477–1487. doi: 10.1007/s00406-022-01532-3, 36574032

[ref142] Rusconi F. Grillo B. Ponzoni L. Bassani S. Toffolo E. Paganini L. . (2016). LSD1 modulates stress-evoked transcription of immediate early genes and emotional behavior. Proc. Natl. Acad. Sci. USA 113, 3651–3656. doi: 10.1073/pnas.1511974113, 26976584 PMC4822633

[ref143] Sauer J. F. Bartos M. (2022). Disrupted-in-schizophrenia-1 is required for normal pyramidal cell–interneuron communication and assembly dynamics in the prefrontal cortex. eLife 11:79471. doi: 10.7554/eLife.79471, 36239988 PMC9566853

[ref144] Schafer D. P. Lehrman E. K. Kautzman A. G. Koyama R. Mardinly A. R. Yamasaki R. . (2012). Microglia sculpt postnatal neural circuits in an activity and complement-dependent manner. Neuron 74, 691–705. doi: 10.1016/j.neuron.2012.03.026, 22632727 PMC3528177

[ref145] Schwarcz R. Bruno J. P. Muchowski P. J. Wu H. Q. (2012). Kynurenines in the mammalian brain: when physiology meets pathology. Nat. Rev. Neurosci. 13, 465–477. doi: 10.1038/nrn3257, 22678511 PMC3681811

[ref146] Seifert T. Brassard P. Wissenberg M. Rasmussen P. Nordby P. Stallknecht B. . (2010). Endurance training enhances BDNF release from the human brain. Am. J. Phys. Regul. Integr. Comp. Phys. 298, R372–R377. doi: 10.1152/ajpregu.00525.2009, 19923361

[ref147] Sekar A. Bialas A. R. De Rivera H. Davis A. Hammond T. R. Kamitaki N. . (2016). Schizophrenia risk from complex variation of complement component 4. Nature 530, 177–183. doi: 10.1038/nature16549, 26814963 PMC4752392

[ref148] Selles M. C. Fortuna J. T. S. de Faria Y. P. R. Siqueira L. D. Lima-Filho R. Longo B. M. . (2023). Oxytocin attenuates microglial activation and restores social and non-social memory in APP/PS1 Alzheimer model mice. iScience 26:106545. doi: 10.1016/j.isci.2023.106545, 37128547 PMC10148027

[ref149] Sellgren C. M. Gracias J. Watmuff B. Biag J. D. Thanos J. M. Whittredge P. B. . (2019). Increased synapse elimination by microglia in schizophrenia patient-derived models of synaptic pruning. Nat. Neurosci. 22, 374–385. doi: 10.1038/s41593-018-0334-7, 30718903 PMC6410571

[ref150] Shigemoto-Mogami Y. Hoshikawa K. Goldman J. E. Sekino Y. Sato K. (2014). Microglia enhance neurogenesis and oligodendrogenesis in the early postnatal subventricular zone. J. Neurosci. 34, 2231–2243. doi: 10.1523/JNEUROSCI.1619-13.2014, 24501362 PMC3913870

[ref151] Shiina A. Kanahara N. Sasaki T. Oda Y. Hashimoto T. Hasegawa T. . (2015). An open study of sulforaphane-rich broccoli sprout extract in patients with schizophrenia. Clin. Psychopharmacol. Neurosci. 13, 62–67. doi: 10.9758/cpn.2015.13.1.62, 25912539 PMC4423155

[ref152] Simon-O’Brien E. Gauthier D. Riban V. Verleye M. (2016). Etifoxine improves sensorimotor deficits and reduces glial activation, neuronal degeneration, and neuroinflammation in a rat model of traumatic brain injury. J. Neuroinflammation 13:203. doi: 10.1186/s12974-016-0687-3, 27565146 PMC5002207

[ref153] Steullet P. Cabungcal J. H. Monin A. Dwir D. O’Donnell P. Cuenod M. . (2016). Redox dysregulation, neuroinflammation, and NMDA receptor hypofunction: a “central hub” in schizophrenia pathophysiology? Schizophr. Res. 176:21. doi: 10.1016/j.schres.2014.06.021 PMC4282982 25000913

[ref154] Stevens B. Allen N. J. Vazquez L. E. Howell G. R. Christopherson K. S. Nouri N. . (2007). The classical complement Cascade mediates CNS synapse elimination. Cell 131, 1164–1178. doi: 10.1016/j.cell.2007.10.036, 18083105

[ref155] Strzelecki D. Podgórski M. Kałuzyńska O. Gawlik-Kotelnicka O. Stefańczyk L. Kotlicka-Antczak M. . (2015). Supplementation of antipsychotic treatment with sarcosine - G1yT1 inhibitor - causes changes of glutamatergic 1NMR spectroscopy parameters in the left hippocampus in patients with stable schizophrenia. Neurosci. Lett. 606, 7–12. doi: 10.1016/j.neulet.2015.08.039, 26306650

[ref156] Sun W. Poschmann J. Cruz-Herrera del Rosario R. Parikshak N. N. Hajan H. S. Kumar V. . (2016). Histone Acetylome-wide association study of autism Spectrum disorder. Cell 167, 1385–1397.e11. doi: 10.1016/j.cell.2016.10.031, 27863250

[ref157] Swathy B. Banerjee M. (2017). Understanding epigenetics of schizophrenia in the backdrop of its antipsychotic drug therapy. Epigenomics 9, 721–736. doi: 10.2217/epi-2016-0106, 28470099

[ref158] Szuhany K. L. Bugatti M. Otto M. W. (2015). A meta-analytic review of the effects of exercise on brain-derived neurotrophic factor. J. Psychiatr. Res. 60, 56–64. doi: 10.1016/j.jpsychires.2014.10.003, 25455510 PMC4314337

[ref159] Tang G. Gudsnuk K. Kuo S. H. Cotrina M. L. Rosoklija G. Sosunov A. . (2014). Loss of mTOR-dependent macroautophagy causes autistic-like synaptic pruning deficits. Neuron 83, 1131–1143. doi: 10.1016/j.neuron.2014.07.040, 25155956 PMC4159743

[ref160] Thomas K. T. Zakharenko S. S. (2021). MicroRNAs in the onset of schizophrenia. Cells 10:2679. doi: 10.3390/cells10102679, 34685659 PMC8534348

[ref161] Tikka T. Fiebich B. L. Goldsteins G. Keinänen R. Koistinaho J. (2001). Minocycline, a tetracycline derivative, is neuroprotective against excitotoxicity by inhibiting activation and proliferation of microglia. J. Neurosci. 21, 2580–2588. doi: 10.1523/JNEUROSCI.21-08-02580.2001, 11306611 PMC6762519

[ref162] Tilleux S. Hermans E. (2007). Neuroinflammation and regulation of glial glutamate uptake in neurological disorders. J. Neurosci. Res. 85, 2059–2070. doi: 10.1002/jnr.21325, 17497670

[ref163] Toader C. Serban M. Munteanu O. Covache-Busuioc R.-A. Enyedi M. Ciurea A. V. . (2025). From synaptic plasticity to neurodegeneration: BDNF as a transformative target in medicine. Int. J. Mol. Sci. 26:4271. doi: 10.3390/ijms26094271, 40362507 PMC12071950

[ref164] Tsai G. Lin P.-Y. (2010). Strategies to enhance N-methyl-D-aspartate receptor-mediated neurotransmission in schizophrenia, a critical review and Meta-analysis. Curr. Pharm. Des. 16, 522–537. doi: 10.2174/138161210790361452, 19909229

[ref165] Valles-Colomer M. Falony G. Darzi Y. Tigchelaar E. F. Wang J. Tito R. Y. . (2019). The neuroactive potential of the human gut microbiota in quality of life and depression. Nat. Microbiol. 4, 623–632. doi: 10.1038/s41564-018-0337-x, 30718848

[ref166] Weinberger D. R. (1987). Implications of Normal brain development for the pathogenesis of schizophrenia. Arch. Gen. Psychiatry 44:660. doi: 10.1001/archpsyc.1987.01800190080012, 3606332

[ref167] Wonodi I. Schwarcz R. (2011). Cortical kynurenine pathway metabolism: a novel target for cognitive enhancement in schizophrenia. Schizophr. Bull. 36, 211–218. doi: 10.1093/schbul/sbq137 PMC2833131 20147364

[ref168] Wulff K. Gatti S. Wettstein J. G. Foster R. G. (2010). Sleep and circadian rhythm disruption in psychiatric and neurodegenerative disease. Nat. Rev. Neurosci. 11, 589–599. doi: 10.1038/nrn2868, 20631712

[ref169] Wykes T. Huddy V. Cellard C. McGurk S. R. Czobor P. (2011). A meta-analysis of cognitive remediation for schizophrenia: methodology and effect sizes. Am. J. Psychiatry 168, 472–485. doi: 10.1176/appi.ajp.2010.10060855, 21406461

[ref170] Yang Y. Liu Y. Wang G. Hei G. Wang X. Li R. . (2019). Brain-derived neurotrophic factor is associated with cognitive impairments in first-episode and chronic schizophrenia. Psychiatry Res. 273, 528–536. doi: 10.1016/j.psychres.2019.01.051, 30710808

[ref171] Yang J. Yang X. Tang K. (2022). Interneuron development and dysfunction. FEBS J. 289, 2318–2336. doi: 10.1111/febs.15872, 33844440

[ref172] Ye L. Huang Y. Zhao L. Li Y. Sun L. Zhou Y. . (2013). IL-1β and TNF-α induce neurotoxicity through glutamate production: a potential role for neuronal glutaminase. J. Neurochem. 125, 897–908. doi: 10.1111/jnc.12263, 23578284 PMC3747774

[ref173] Yeh F. L. Hansen D. V. Sheng M. (2017). TREM2, microglia, and neurodegenerative diseases. Trends Mol. Med. 23, 512–533. doi: 10.1016/j.molmed.2017.03.008, 28442216

[ref174] Yeh H. Ikezu T. (2019). Transcriptional and epigenetic regulation of microglia in health and disease. Trends Mol. Med. 25, 96–111. doi: 10.1016/j.molmed.2018.11.004, 30578089 PMC6377292

[ref175] Yirmiya R. Goshen I. (2011). Immune modulation of learning, memory, neural plasticity and neurogenesis. Brain Behav. Immun. 25, 181–213. doi: 10.1016/j.bbi.2010.10.015, 20970492

[ref176] Yong V. W. Wells J. Giuliani F. Casha S. Power C. Metz L. M. (2004). The promise of minocycline in neurology. Lancet Neurol. 3, 744–751. doi: 10.1016/S1474-4422(04)00937-8, 15556807

[ref177] Yoshii A. Constantine-Paton M. (2007). BDNF induces transport of PSD-95 to dendrites through PI3K-AKT signaling after NMDA receptor activation. Nat. Neurosci. 10, 702–711. doi: 10.1038/nn1903, 17515902

[ref178] Yousaf M. Chang D. Liu Y. Liu T. Zhou X. (2022). Neuroprotection of Cannabidiol, its synthetic derivatives and combination preparations against microglia-mediated Neuroinflammation in neurological disorders. Molecules 27:4961. doi: 10.3390/molecules27154961, 35956911 PMC9370304

[ref179] Yrjänheikki J. Tikka T. Keinänen R. Goldsteins G. Chan P. H. Koistinaho J. (1999). A tetracycline derivative, minocycline, reduces inflammation and protects against focal cerebral ischemia with a wide therapeutic window. Proc. Natl. Acad. Sci. USA 96, 13496–13500. doi: 10.1073/pnas.96.23.13496, 10557349 PMC23976

[ref180] Yu Y. Shen Q. Lai Y. Park S. Y. Ou X. Lin D. . (2018). Anti-inflammatory effects of curcumin in microglial cells. Front. Pharmacol. 9:386. doi: 10.3389/fphar.2018.00386, 29731715 PMC5922181

[ref181] Yuan L. Liu S. Bai X. Gao Y. Liu G. Wang X. . (2016). Oxytocin inhibits lipopolysaccharide-induced inflammation in microglial cells and attenuates microglial activation in lipopolysaccharide-treated mice. J. Neuroinflammation 13:77. doi: 10.1186/s12974-016-0541-7, 27075756 PMC4831099

[ref182] Zhang J. Zheng Y. Luo Y. Du Y. Zhang X. Fu J. (2019). Curcumin inhibits LPS-induced neuroinflammation by promoting microglial M2 polarization via TREM2/ TLR4/ NF-κB pathways in BV2 cells. Mol. Immunol. 116, 29–37. doi: 10.1016/j.molimm.2019.09.020, 31590042

[ref183] Zhao N. Francis N. L. Calvelli H. R. Moghe P. V. (2020). Microglia-targeting nanotherapeutics for neurodegenerative diseases. APL Bioeng. 4:030902. doi: 10.1063/5.0013178, 32923843 PMC7481010

[ref184] Zheng P. Zeng B. Liu M. Chen J. Pan J. Han Y. . (2019). The gut microbiome from patients with schizophrenia modulates the glutamate-glutamine-GABA cycle and schizophrenia-relevant behaviors in mice. Sci. Adv. 5:eaau8317. doi: 10.1126/sciadv.aau8317, 30775438 PMC6365110

[ref185] Zhou Z. Li Y. Peng R. Shi M. Gao W. Lei P. . (2024). Progesterone induces neuroprotection associated with immune/inflammatory modulation in experimental traumatic brain injury. Neuroreport 35, 352–360. doi: 10.1097/WNR.0000000000002013, 38526937 PMC10965124

[ref186] Zhou T. Zhang S. Zhou Y. Lai S. Chen Y. Geng Y. . (2021). Curcumin alleviates imiquimod-induced psoriasis in progranulin-knockout mice. Eur. J. Pharmacol. 909:174431. doi: 10.1016/j.ejphar.2021.174431, 34428436

