# Correction: Is it possible to prevent excessive synaptic pruning in schizophrenia? Possibilities and limitations

**DOI:** 10.3389/fnsyn.2026.2007021

**Published:** 2026-09-30

**Authors:** Agnieszka Pawlak, Jakub Stefanowicz, Zofia Kotkowska, Agata Gabryelska, Marcin Sochal, Filip Napieraj, Magdalena Kotlicka-Antczak, Dominik Strzelecki

**Affiliations:** 1 Department of Affective and Psychotic Disorders, Medical University of Łódz, Łódz, Poland; 2 Department of Sleep Medicine and Metabolic Disorders, Medical University of Łódz, Łódz, Poland; 3 Medical University of Warsaw, Warsaw, Poland; 4 Department of Child and Adolescent Psychiatry, Medical University of Łódz, Łódz, Poland

**Keywords:** schizophrenia, synaptic pruning, microglia, C4A, BDNF, kynurenine pathway, neuroprotection, psychosis prevention

In the published article, in the **Funding** statement, the funder “Foundation for Polish Science (FNP)” was erroneously omitted. The corrected Funding statement reads:

“The author(s) declared that financial support was received for this work and/or its publication. This work was supported by statutory funds of the Department of Affective and Psychotic Disorders, Medical University of Lodz, Poland. This work was also supported by the Foundation for Polish Science (FNP).”

The original version of this article has been updated.

